# Single‐Molecule Magnets DyM_2_N@C_80_ and Dy_2_MN@C_80_ (M=Sc, Lu): The Impact of Diamagnetic Metals on Dy^3+^ Magnetic Anisotropy, Dy⋅⋅⋅Dy Coupling, and Mixing of Molecular and Lattice Vibrations

**DOI:** 10.1002/chem.201904879

**Published:** 2020-02-06

**Authors:** Lukas Spree, Christin Schlesier, Aram Kostanyan, Rasmus Westerström, Thomas Greber, Bernd Büchner, Stanislav M. Avdoshenko, Alexey A. Popov

**Affiliations:** ^1^ Leibniz Institute for Solid State and Materials Research (IFW Dresden) 01069 Dresden Germany; ^2^ Physik-Institut der Universität Zürich Winterthurerstr. 190 8057 Zürich Switzerland; ^3^ The Division of Synchrotron Radiation Research Lund University 22100 Lund Sweden

**Keywords:** dysprosium, exchange interactions, fullerenes, lutetium, magnetic properties, phonons

## Abstract

The substitution of scandium in fullerene single‐molecule magnets (SMMs) DySc_2_N@C_80_ and Dy_2_ScN@C_80_ by lutetium has been studied to explore the influence of the diamagnetic metal on the SMM performance of dysprosium nitride clusterfullerenes. The use of lutetium led to an improved SMM performance of DyLu_2_N@C_80_, which shows a higher blocking temperature of magnetization (*T*_B_=9.5 K), longer relaxation times, and broader hysteresis than DySc_2_N@C_80_ (*T*_B_=6.9 K). At the same time, Dy_2_LuN@C_80_ was found to have a similar blocking temperature of magnetization to Dy_2_ScN@C_80_ (*T*_B_=8 K), but substantially different interactions between the magnetic moments of the dysprosium ions in the Dy_2_MN clusters. Surprisingly, although the intramolecular dipolar interactions in Dy_2_LuN@C_80_ and Dy_2_ScN@C_80_ are of similar strength, the exchange interactions in Dy_2_LuN@C_80_ are close to zero. Analysis of the low‐frequency molecular and lattice vibrations showed strong mixing of the lattice modes and endohedral cluster librations in *k*‐space. This mixing simplifies the spin–lattice relaxation by conserving the momentum during the spin flip and helping to distribute the moment and energy further into the lattice.

## Graphical abstract

**What a mix**! The diamagnetic metal in DyM_2_N@C_80_ and Dy_2_MN@C_80_ (M=Lu or Sc) has a considerable influence on the single‐molecule magnetism of clusterfullerenes. DyLu_2_N@C_80_ has a higher blocking temperature of magnetization than DySc_2_N@C_80_, whereas exchange coupling in Dy_2_LuN@C_80_ is much weaker than in Dy_2_ScN@C_80_. The different masses of lutetium and scandium also alter the mixing of local cluster vibrations and lattice modes, and hence have a strong impact on the spin–lattice relaxation (see figure).

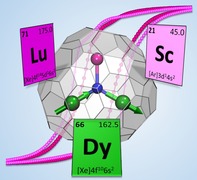

## Introduction

Enclosing lanthanide ions within the fullerene cage is a versatile route to a variety of molecular magnets.^1^ In particular, when non‐metal atoms (C, N, O, S) are captured by the carbon cage together with lanthanides, the strong ionic interactions emerging in such endohedral clusterfullerenes may lead to a large magnetic anisotropy.^2^ In addition, different magnetic states can emerge from the intramolecular interactions of lanthanide ions in clusterfullerenes.2a, 2e, 3 This combination of properties made lanthanide‐clusterfullerenes promising candidates for single‐molecule magnets (SMMs). Single‐molecule magnetism is a phenomenon involving the slow relaxation of magnetization in molecules with a bistable magnetic ground state and has been a hot topic in the field of molecular magnetism during the last decades.^4^ Indeed, some lanthanide‐clusterfullerenes exhibit single‐molecule magnetism,^5^ which is most robust in dysprosium‐clusterfullerenes.1c, 2d–2f, 3a, 6

Nitride clusterfullerenes (NCFs) with the composition (M^3+^)_3_N^3−^@C_2*n*_^6−^, and in particular the species with C_2*n*_=C_80_‐*I_h_* (*I_h_* denotes the symmetry of the carbon cage) and M=Sc, Y, or heavy lanthanides (Gd–Lu), show the largest synthetic yield and are therefore the most studied clusterfullerenes to date.1a, 1b, 7 The structure of the trimetal nitride cluster, with N^3−^ in its center and three M^3+^ ions located at the vertices of the triangle with rather short M−N bonds, offers a convenient platform to create a variety of molecular magnets with divergent properties. First of all, the nitride ion at a distance of only 2.0–2.2 Å from the lanthanide2d, 6b, 8 generates a strong axial ligand field (LF), which leads to an easy‐axis magnetic anisotropy for cerium, praseodymium, neodymium, terbium, dysprosium, and holmium ions, and an easy‐plane anisotropy for erbium and thulium ions2b (note that we use the term “ligand field” instead of the more commonly used “crystal field” to avoid unnecessary connotations to intermolecular interactions in crystals). The strong axial LF also ensures that *m_J_* is a good quantum number, at least for several lowest‐energy LF states. In particular, the lowest‐energy Kramers doublets for Dy^3+^ in dysprosium‐scandium NCFs are essentially pure *m_J_* states, with the high‐spin *J_z_*=±15/2 states as the ground Kramers doublet with the magnetic moment oriented along the Dy−N bond.2a, 2c The purity of the *J_z_* states in terms of *m_J_* composition ensures that the magnetic system is weakly susceptible to external perturbations such as those introduced by dipolar magnetic fields from neighboring molecules or molecular and lattice vibrations.

**Figure 1:**
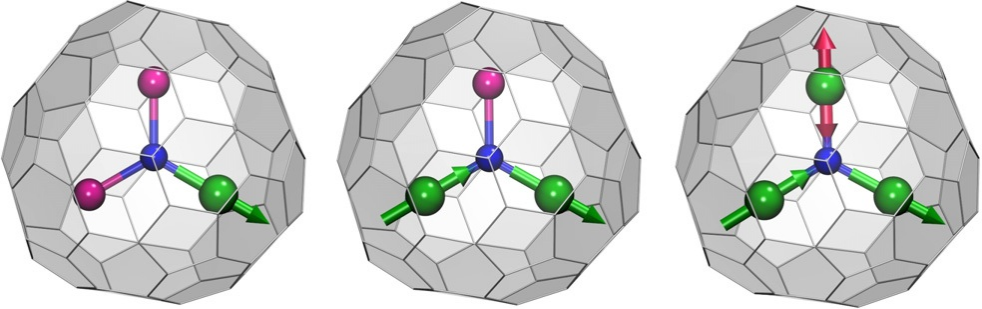
Molecular structures of Dy_*x*_M_3−*x*_N@C_80_‐*I_h_* nitride clusterfullerenes (M=Sc or Lu; *x=*1–3) and schematic illustration of the coupling of magnetic moments. Dy is green, M is magenta, N is blue, C is light gray. The magnetic moments of Dy ions are visualized as green or red arrows. In DyM_2_N@C_80_ (left), the magnetic moment is aligned along the Dy−N bond. In the magnetic ground state of Dy_2_MN@C_80_ (middle), the magnetic moments of the two Dy ions are coupled ferromagnetically. In Dy_3_N@C_80_ (right), the ground magnetic state is frustrated, and the magnetic moment of the Dy ion illustrated with a red arrow can switch between two isoenergetic orientations without changing the orientation of the two other moments.

Another advantage that lanthanide NCFs offer for tuning magnetic properties is the possibility of combining different metals within one molecule to give the so‐called mixed‐metal NCFs.5a, 8c, 9 The importance of this can be best illustrated with the dysprosium‐scandium NCFs Dy_*x*_Sc_3−*x*_N@C_80_‐*I_h_* (*x=*1–3) as an example.3a As Sc^3+^ is diamagnetic, this series essentially allows analysis of how two or three dysprosium ions interact magnetically and the effect of these interactions on SMM behavior. DySc_2_N@C_80_‐*I_h_* was the first endohedral metallofullerene (EMF) proven to be a SMM.6b, 6d At temperatures below 7 K, the molecule exhibits magnetic hysteresis with the abrupt drop of the magnetization in zero magnetic field ascribed to the quantum tunneling of magnetization (QTM), which is typical for single‐ion magnets. Dy_2_ScN@C_80_‐*I_h_* shows magnetic hysteresis below 8 K without fast QTM relaxation in zero field, which is explained by ferromagnetic exchange and dipolar coupling between the non‐collinear magnetic moments of the two dysprosium ions in the Dy_2_ScN cluster (Figure 1), thus creating an additional barrier and preventing zero‐field QTM.3a The temperature dependence of the magnetization relaxation times in Dy_2_ScN@C_80_‐*I_h_* revealed a high barrier of 1735 K, because of the Orbach relaxation via the fifth Kramers doublet, in good agreement with CASSCF calculations.2d Similar ferromagnetic interactions between dysprosium ions are also present in Dy_3_N@C_80_‐*I_h_*, but the triangular arrangement of the dysprosium ions forbids simultaneous realization of ferromagnetic coupling for all three Dy⋅⋅⋅Dy contacts resulting in a frustrated ground state (Figure 1) with faster relaxation of magnetization than in the mono‐ and dinuclear analogues.2a, 3a Thus, due to different cluster compositions and intramolecular Dy⋅⋅⋅Dy interactions, DySc_2_N@C_80_‐*I_h_*, Dy_2_ScN@C_80_‐*I_h_*, and Dy_3_N@C_80_‐*I_h_* exhibit substantially different SMM behavior at low temperatures.

It is natural to consider that the magnetic properties of the NCFs in the Dy_*x*_Sc_3−*x*_N@C_80_‐*I_h_* (*x=*1–3) series discussed above are determined by the Dy^3+^ ions, with the diamagnetic Sc^3+^ ions acting just as placeholders, which are needed to keep the trimetallic cluster composition. However, in this work, we shed more light on the role that the diamagnetic metal can play in the SMM properties of NCFs. For this, we chose lutetium as a diamagnetic lanthanide, the ionic radius (*R* ^3+^=0.86 Å) of which is noticeably larger than that of scandium (*R* ^3+^=0.75 Å). Yet, as we reported recently, DyLu_2_N@C_80_‐*I_h_* and Dy_2_LuN@C_80_‐*I_h_* can still be synthesized, albeit in lower yields than the dysprosium‐scandium analogues.8c The larger size of Lu^3+^ leads to changes in the internal structure of the trimetallic nitride cluster, and in particular results in shorter Dy−N bonds than in the dysprosium‐scandium NCFs. The shortening of the Dy−N bonds may affect the magnetic anisotropy and the strength of the intramolecular Dy⋅⋅⋅Dy coupling. Furthermore, lutetium is much heavier than scandium, which changes the low‐frequency part of the vibrational spectrum. Because the relaxation of magnetization involves spin–phonon interactions, alterations of molecular vibrations may also result in changes in the SMM properties. Thus, the goal of this work was to study a possible influence of these factors on the SMM properties of the DyM_2_N@C_80_‐*I_h_* and Dy_2_MN@C_80_‐*I_h_* NCFs.

## Results and Discussion

### Magnetization behavior of DyLu_2_N@C_80_ and Dy_2_LuN@C_80_

The mixed‐metal dysprosium‐lutetium NCFs with a C_80_‐*I_h_* fullerene cage (for clarity, the symmetry designation will be omitted hereafter) were obtained by arc‐discharge synthesis and separated by recycling HPLC as reported earlier.8c Similar retention behavior of Dy_3_N@C_80_, Dy_2_LuN@C_80_, DyLu_2_N@C_80_, and Lu_3_N@C_80_ substantially complicated the separation of the individual compounds. However, a compositional purity exceeding 90–95 % could be achieved for Dy_2_LuN@C_80_, as verified by MS analysis. Due to the very similar retention behavior, the separation of DyLu_2_N@C_80_ from Lu_3_N@C_80_ was not possible, and the relative content of the two NCFs in the studied sample was around 1:1.35. Because Lu_3_N@C_80_ is diamagnetic, its presence in the sample does not lead to strong changes in the magnetic properties of DyLu_2_N@C_80_. A possible influence of dilution on the quantum tunneling of magnetization will be specifically considered below.

**Figure 2:**
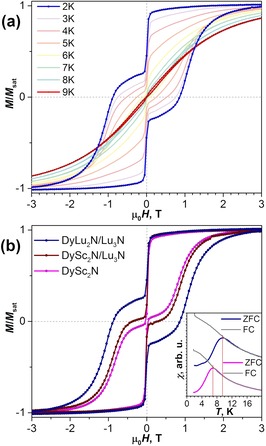
(a) Magnetization curves of the DyLu_2_N@C_80_/Lu_3_N@C_80_ sample measured between 2 and 9 K (average sweep rate 2.9 mT s^−1^). (b) Magnetization curves of DyLu_2_N@C_80_/Lu_3_N@C_80_ measured at 2 K and compared with those of DySc_2_N@C_80_ and DySc_2_N@C_80_ diluted with Lu_3_N@C_80_ in a 1:1 ratio. The inset in (b) shows the determination of the blocking temperature, *T*_B_, from the temperature dependence of the magnetic susceptibility, *χ* (temperature sweep rate of 5 K min^−1^).

**Figure 3:**
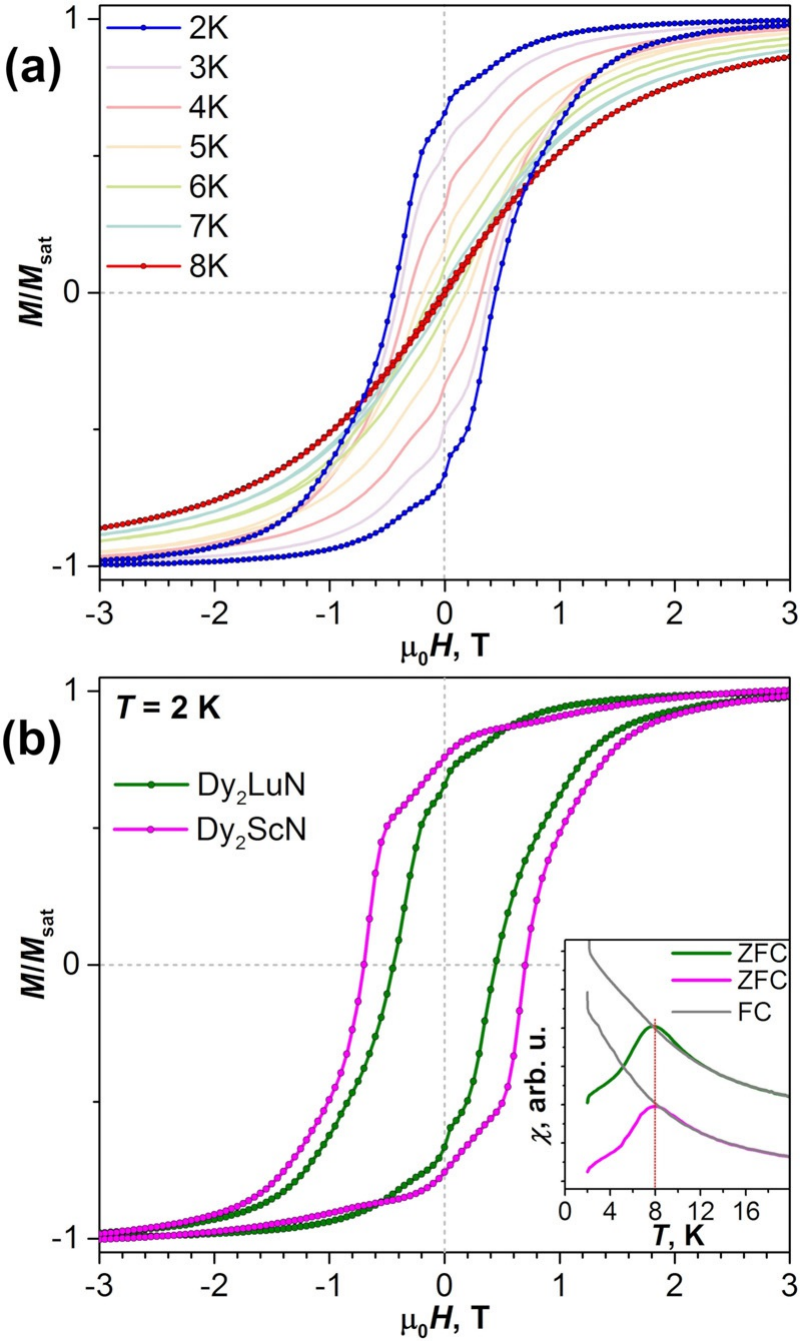
(a) Magnetization curves of the Dy_2_LuN@C_80_ sample measured between 2 and 8 K (average sweep rate 2.9 mT s^−1^). (b) Magnetization curve of Dy_2_LuN@C_80_ measured at 2 K and compared with that of Dy_2_ScN@C_80_. The inset in (b) shows the determination of the blocking temperature, *T*_B_, from the temperature dependence of the magnetic susceptibility, *χ* (temperature sweep rate of 5 K min^−1^).

The magnetization curves of DyLu_2_N@C_80_ and Dy_2_LuN@C_80_, measured by SQUID (superconducting quantum interference device) magnetometry, are shown in Figures 2 and 3, respectively. DyLu_2_N@C_80_ exhibits hysteresis up to 9 K at a sweep rate of 3 mT s^−1^ (Figure 2 a). The “butterfly” shape of the hysteresis curves points to efficient zero‐field relaxation by QTM similar to that observed in DySc_2_N@C_80_ (Figure 2 b).6d However, in the latter, the opening of the hysteresis is narrower and the QTM induces complete loss of magnetization at zero field, whereas in DyLu_2_N@C_80_, upon crossing zero‐field, the magnetization drops to around 30 % of the saturation magnetization value, resulting in a coercivity of 0.9 T at 2 K. Recently, we showed that the QTM in DySc_2_N@C_80_ is strongly affected by dilution in the diamagnetic matrix, including dilution with Lu_3_N@C_80_.6b To ensure that the difference in the QTM is intrinsic for the two NCFs and is not caused by the dilution of DyLu_2_N@C_80_ with Lu_3_N@C_80_, in Figure 2 b we also show the magnetization curve of DySc_2_N@C_80_ diluted with Lu_3_N@C_80_ in a ratio of 1:1. This magnetic dilution indeed reduces the QTM step in DySc_2_N@C_80_ slightly, but the changes do not reach the magnitude observed for DyLu_2_N@C_80_. Thus, it can be concluded that the zero‐field QTM relaxation of magnetization in DyLu_2_N@C_80_ is slower than in DySc_2_N@C_80_. Furthermore, the blocking temperature of magnetization, *T*_B_, defined as the temperature of peak magnetic susceptibility measured at 0.2 T for the sample cooled in zero field, is higher for DyLu_2_N@C_80_ (*T*_B_=9.5 K) than for DySc_2_N@C_80_ (*T*_B_=6.9 K). Note that the magnetic dilution does not affect the *T*_B_ value,6b and thus the different SMM properties of DyLu_2_N@C_80_ and DySc_2_N@C_80_ cannot be caused by the presence of Lu_3_N@C_80_ in the former. To conclude, DyLu_2_N@C_80_ was found to be a stronger SMM than DySc_2_N@C_80_. The substitution of scandium by lutetium in DyM_2_N@C_80_ leads to slower QTM relaxation, slower in‐field relaxation, and a broader magnetic hysteresis with remanence.

For Dy_2_MN@C_80_, the influence of the diamagnetic metal on the SMM behavior appears to be weaker than for DyM_2_N@C_80_. Similarly to Dy_2_ScN@C_80_, Dy_2_LuN@C_80_ exhibits open hysteresis without a pronounced QTM step (Figure 3 a). Its blocking temperature of magnetization, *T*_B_=8 K, is the same as that of Dy_2_ScN@C_80_.2d However, at 2 K, the magnetic hysteresis of Dy_2_LuN@C_80_ is narrower (Figure 3 b) and the coercive field of 0.4 T is smaller than that of Dy_2_ScN@C_80_ (0.7 T). Thus, the substitution of scandium by lutetium narrows the magnetic hysteresis but does not change the temperature scale of the slow relaxation. Nevertheless, as we show below, the temperature dependence of relaxation times reveals that the mechanisms of the relaxation of magnetization in Dy_2_LuN@C_80_ and Dy_2_ScN@C_80_ are different.

### Relaxation times of magnetization in DyM_2_N@C_80_ and Dy_2_MN@C_80_

The opening of magnetic hysteresis in the magnetization curves of molecular magnets indicates that the magnetization attains its equilibrium value slower than the rate of the magnetic field sweep. The slow relaxation of magnetization is a key characteristic of SMMs and needs to be understood in detail. The relaxation of magnetization requires energy exchange between the spin system and a thermal bath, which is mediated by the phonon system. The spin–phonon interaction is crucial for such an energy exchange, and the role of vibrational degrees of freedom becomes paramount. Several mechanisms of phonon‐mediated spin relaxation have been recognized in studies of paramagnetic salts and adapted for SMMs.

A direct mechanism implies a single‐phonon process in which phonon frequency matches the energy difference between two opposite spins. Because a magnetic field increases the energy gap between the opposite spins, and the phonon density at near‐zero frequency is low and increases with frequency, the relaxation due to a direct mechanism accelerates in a magnetic field according to Equation (1)

(1) $$\tau_{M,dir}^{-1}\left(H\right)=A_{1}H^{4}T+A_{2}H^{2}T$$

in which *τ*_M_ is the magnetization relaxation time, *H* is the magnetic field, with two terms describing the relaxation of a Kramers ion in the absence (ca. *H* ^4^) and in the presence (ca. *H* ^2^) of hyperfine interactions, and *A*_1_ and *A*_2_ are fitting parameters. Thus, the relaxation rate scales linearly with temperature, $\tau_{M,dir}^{-1}∼T$ . However, when at low temperature the phonon density may be very low, the energy exchange between the phonon system and the bath can become the limiting step. This effect, known as a phonon bottleneck, can change the temperature dependence to $\tau_{M,dir}^{-1}∼T^{2}$ .

The Raman mechanism implies a spin flip through the absorption and emission of two phonons, with the frequency difference being equal to the energy gap between the opposite spins. As the frequencies can be much higher than the Zeeman energy, and the phonon density increases with frequency, the Raman mechanism is more efficient than the direct mechanism once the temperature is sufficiently high to ensure sufficient phonon population. The original consideration with only acoustic phonons in the Debye model gave the power‐law temperature dependence expressed by Equation (2)

(2) $$\tau_{M,Ram}^{-1}\left(T\right)=CT^{n}$$

in which *n=*9 for Kramers ions and *n=*7 for non‐Kramers ions, and *C* is the fitting parameter.^10^ However, if optical phonons are also included in the model, powers of 6, 5, and even lower can be expected.^11^

A special case of the Raman mechanism, in which the absorbed phonon energy corresponds to the real excited spin state, is known as the Orbach mechanism. The temperature dependence of the relaxation rate under the Orbach mechanism has an Arrhenius form, expressed by Equation (3)

(3) $$\tau_{M,Orb}^{-1}\left(T\right)=\tau_{0}^{-1}exp\left(-U^{eff}/T\right)$$

in which *U* ^eff^ is the effective barrier (corresponding to the energy of the excited magnetic state) and *τ*_0_ is the attempt time.^10^

Finally, the QTM is a ubiquitous and characteristic relaxation mechanism of SMMs. In QTM, the spin flips to the opposite direction without energy transfer. As such, it should not show temperature dependence. The key condition for QTM is the energy matching of the opposite spin levels, and therefore the application of a magnetic field can quench the QTM when Zeeman splitting becomes large enough.

It has become common practice to analyze the relaxation of magnetization in SMMs as a combination of these processes.4f, 12 Characteristic temperature and field dependencies of relaxation times allow identification of the prevailing relaxation mechanism. Usually, the direct mechanism is the most important at the lowest temperatures of a few K, at somewhat higher temperatures the Raman mechanism becomes dominant, and with further increases in temperature, the Orbach mechanism involving LF excited states takes over. Neither the direct nor Raman mechanism would show an Arrhenius temperature dependence, so the latter usually serves as an indication of the Orbach mechanism. However, back in the 1960s, Klemens^13^ and others^14^ argued that a localized vibrational mode can cause an Arrhenius temperature dependence with *U* ^eff^ corresponding to the frequency of the mode. Furthermore, it was shown that the direct mechanism can also demonstrate Arrhenius behavior in some conditions.11a, 15

The relaxation times of magnetization in DyLu_2_N@C_80_ and Dy_2_LuN@C_80_ in this work were determined at different temperatures by magnetizing the sample to saturation, quickly sweeping the magnetic field to zero or any other required field value, and then following the decay of magnetization with subsequent fitting of the measured decay curves with a stretched exponential (see the Supporting Information for the decay curves and Tables containing all the fitted parameters). Below, the values obtained here are compared with those of DySc_2_N@C_80_ from ref. 6b, whereas the relaxation times in Dy_2_ScN@C_80_ were re‐measured in this work for better consistency.

**Figure 4:**
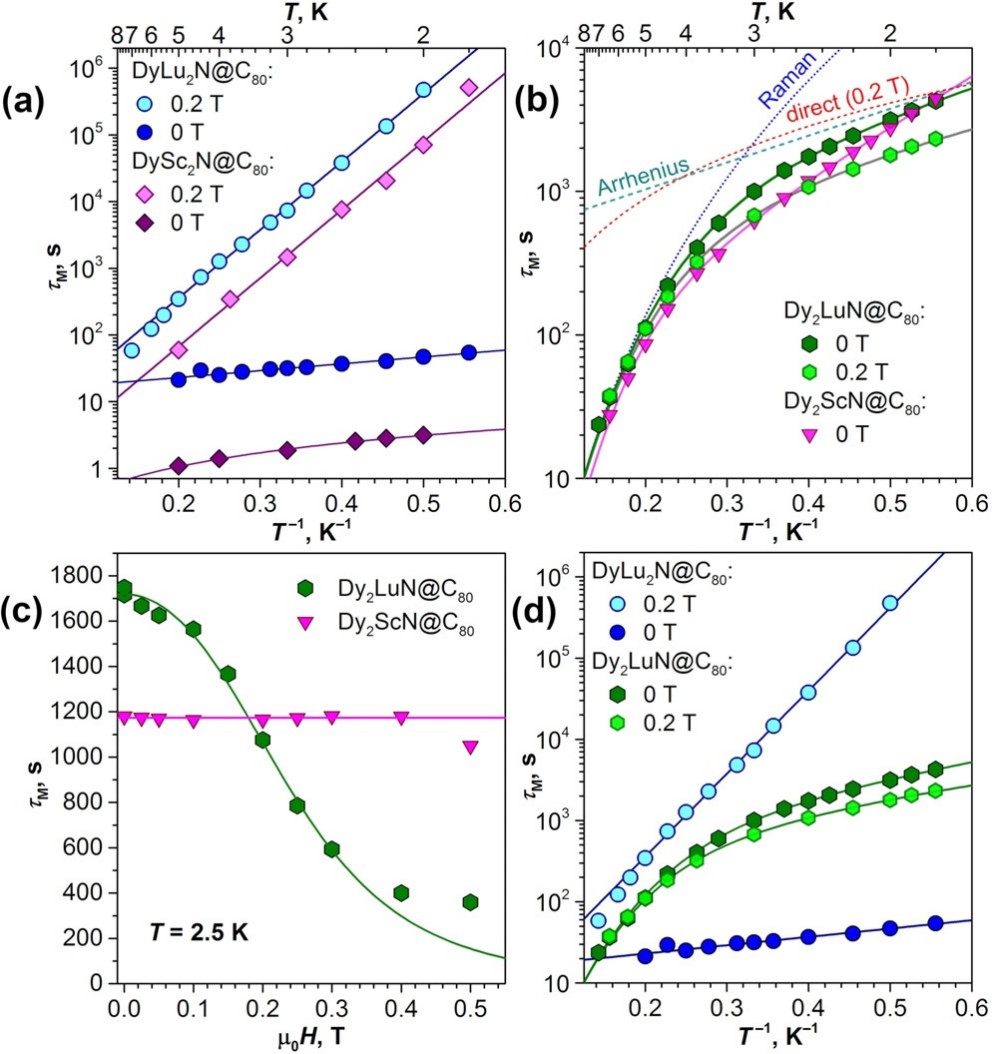
(a) Magnetization relaxation times of DyLu_2_N@C_80_ measured in zero field and in a field of 0.2 T and compared with those of DySc_2_N@C_80_ from ref. 6b (the zero‐field values for DySc_2_N@C_80_ were measured by AC magnetometry). The lines are fits of the Arrhenius behavior. (b) Magnetization relaxation times of Dy_2_LuN@C_80_ measured in zero field and in a field of 0.2 T and compared with the relaxation times of Dy_2_ScN@C_80_ in zero field. The solid lines are total fits with Equations (4) and (5) (0 and 0.2 T, respectively). The contributions of the Arrhenius, Raman, and direct (for 0.2 T) processes are also shown. (c) Field dependence of magnetization relaxation times of Dy_2_LuN@C_80_ and Dy_2_ScN@C_80_ measured at 2.5 K. The green line is the fit of Dy_2_LuN@C_80_ data with Equation (1). (d) Comparison of the magnetization relaxation times of DyLu_2_N@C_80_ and Dy_2_LuN@C_80_.

The zero‐field measurements for DyLu_2_N@C_80_ are complicated by the relatively long stabilization of the field and fast zero‐field QTM. In addition, the decay curves showed two types of behavior: A fast drop of magnetization for around 90 % of the sample, followed by a much slower relaxation of the remaining magnetization. The fit of the decay curves with two stretched exponents gave values ranging from 54±1 s at 1.8 K to 21±6 s at 5 K for the fast process (Figure 4 a), which we assigned to QTM. The abrupt change in the relaxation rate may be caused by the redistribution of dipolar fields in the sample when a significant number of spins flip, the dilution effect of Lu_3_N@C_80_, and may also reflect different relaxation of the molecules with different dysprosium isotopes. In addition, the slow process may also be caused by slow relaxation of the remnant magnetization in the magnet, or the deviation of the real magnetic field from zero. For comparison, *τ*_QTM_ in nondiluted DySc_2_N@C_80_ determined by AC magnetometry in the same temperature range is 1–3 s.6b Overall, we can conclude that the rate of relaxation by the QTM mechanism in DyLu_2_N@C_80_ is slower than in DySc_2_N@C_80_. Note that both compounds show a temperature dependence of the relaxation rate, even in the QTM regime, which may reflect the temperature dependence of the phonon collision rate, as suggested by Chilton and co‐workers,^16^ or result from dipolar intermolecular interactions.

Short relaxation times cannot be measured very reliably by DC magnetometry, and further relaxation measurements were performed for DyLu_2_N@C_80_ in a field of 0.2 T, applied to quench the QTM. Over a range of only 5 K, the in‐field relaxation times in DyLu_2_N@C_80_ vary by almost four orders of magnitude, from 4.7×10^5^ s at 2 K to 58 s at 7 K (Figure 4 a). When plotted in Arrhenius coordinates, the temperature dependence of the relaxation times has a linear form below 5 K. For comparison, the *τ*_M_ values of DySc_2_N@C_80_ are systematically shorter than those of DyLu_2_N@C_80_ by a factor of six, but they also show a linear temperature dependence with a very similar inclination (Figure 4 a). Fitting the relaxation times of DyLu_2_N@C_80_ with Equation (3) gave a *U* ^eff^ value of 24.2±0.7 K and *τ*_0_ of 2.8±0.5 s. The analogous fit for DySc_2_N@C_80_ gave *U* ^eff^=23.6±1 K and *τ*_0_=0.6±0.2 s.6b Thus, both NCFs have essentially identical *U* ^eff^ values and differ only in their attempt times. The reason for the low‐temperature *U* ^eff^ barriers of 24 K in both DyM_2_N@C_80_ molecules is not clear. Due to the very strong LF splitting of dysprosium in the NCFs, the energies of the lowest‐energy excited states exceed hundreds of K (see the discussion of the ab initio calculations below).2a–2d, 2f, 2g In addition, the *τ*_0_ values are many orders of magnitude longer than are usually found for the Orbach mechanism. We tentatively propose that the relaxation of magnetization in SMM EMFs in this temperature range may follow the Raman mechanism with involvement of local vibrations that would also follow Equation (3), but with *U* ^eff^ corresponding to the vibrational frequency.14a, 17 The vibrational density of states in dysprosium NCFs is discussed further below.

The temperature dependence of the relaxation times of Dy_2_LuN@C_80_ measured in zero magnetic field has a curved shape in the log (*τ*_M_) versus *T* ^−1^ plot (Figure 4 b) and can be described well by using a combination of Raman and Arrhenius processes, expressed by Equation (4).

(4) $$\tau_{M}^{-1}\left(T\right)=CT^{n_{1}}+\tau_{0}^{-1}exp\left(-U^{eff}/T\right)$$

The fit of the experimental zero‐field relaxation times of Dy_2_LuN@C_80_ with Equation (4) gives *C*=(1.14±0.28)×10^−6^ s^−1^ K^−5.45^, *n*_1_=5.45±0.15, *τ*_0_=435±50 s, and *U* ^eff^=4.3±0.2 K (Table 1). The Raman mechanism dominates above 4 K, whereas Arrhenius behavior is predominant below 2.5 K, and both mechanisms make comparable contributions in between. As for DyLu_2_N@C_80_, neither *τ*_0_ nor *U* ^eff^ of the Arrhenius part is typical for the standard Orbach relaxation process via ligand‐field excited states. Spin relaxation in Dy_2_ScN@C_80_ below 8 K can be also described by Equation (4) with *C*=(0.51±0.26)×10^−6^ s^−1^ K^−5.99^, *n*_1_=5.99±0.33, *τ*_0_=56±4 s, and *U* ^eff^=8.0±0.1 K (somewhat different values, *τ*_0_=11.9±1.5 s and *U* ^eff^=10.7±0.3 K, reported by us in ref. 2d, were obtained with a smaller data set). Thus, Dy_2_LuN@C_80_ and Dy_2_ScN@C_80_ have similar parameters in the Raman process, but are significantly different in their Arrhenius behavior, as Dy_2_ScN@C_80_ has a two‐fold higher *U* ^eff^ and an eight‐fold shorter *τ*_0_. As a result, although the zero‐field relaxation times of Dy_2_LuN@C_80_ and Dy_2_ScN@C_80_ are very similar at 1.8 K and virtually identical by 7 K, between 2 and 6 K the relaxation of magnetization in Dy_2_LuN@C_80_ is considerably slower.

**Table 1:** Low‐temperature^[a]^ relaxation parameters for DyM_2_N@C_80_ and Dy_2_MN@C_80_ (M=Sc, Lu).

	QTM	Raman		Arrhenius		Direct	
	$\tau_{QTM}^{-1}$	$CT^{n_{1}}$		$\tau_{0}^{-1}exp\left(-U^{eff}/T\right)$		$A\left(H\right)T^{n_{2}}$	
M_3_N@C_80_, field	*τ*_QTM_ [s]	*C* [10^−6^ s^−1^ K^−*n*1^]	*n*_1_	*τ*_0_ [s]	*U* ^eff^ [K]	*A*(*H*) [10^−5^ s^−1^ K^−*n*2^]	*n*_2_
DyLu_2_N, 0 T	20–50	–	–	–	–	–	–
DyLu_2_N, 0.2 T	–	–	–	2.8±0.5	24.2±0.7	–	–
DySc_2_N, 0 T	1–3	–	–	–	–	–	–
DySc_2_N, 0.2 T	–	–	–	0.6±0.2	23.6±1.0	–	–
							
Dy_2_LuN, 0 T	–	1.14±0.28	5.45±0.15	435±50	4.3±0.2	–	–
Dy_2_LuN, 0.2 T	–	1.14±0.28	5.45±0.15	435±50	4.3±0.2	7.59±0.57	1.67±0.09
Dy_2_ScN, 0 T	–	0.51±0.26	5.99±0.33	56±4	8.0±0.1	–	–

[a] Below *T*_B_.

Measurements of magnetization relaxation times in Dy_2_LuN@C_80_ at 2.5 K in different magnetic fields (Figure 4 c) revealed considerable acceleration of the relaxation with increasing field. The temperature dependence of the relaxation times measured in a field of 0.2 T also showed noticeable deviations from zero‐field values below 5 K (Figure 4 b). Such a dependence of *τ*_M_ on the magnetic field is a characteristic of the direct relaxation mechanism [Eq. (1)]. To describe the temperature dependence of the relaxation times measured in a field of 0.2 T, we used Equation (4) with the addition of a term describing the direct process, given by Equation (5)

(5) $$\tau_{M}^{-1}\left(T\right)=CT^{n_{1}}+\tau_{0}^{-1}exp\left(-U^{eff}/T\right)+A\left(H\right)T^{n_{2}}$$

in which *C*, *n*_1_, *τ*_0_, and *U* ^eff^ were fixed to the values determined for zero‐field relaxation.

The fit of the experimental data measured in a field of 0.2 T with Equation (5) gives *A*(*H*)=(7.59±0.57)×10^−5^ s^−1^ K^−1.67^>and *n*_2_=1.67±0.09 (Table 1). The exponent of approximately 1.7 lies between the values expected for normal (*n=*1) and bottleneck (*n=*2) direct processes, and indicates that both are likely to take place. If instead of using the fitting procedure, the *n*_2_ value is fixed to 1 and *A*(*H*) is determined from the field dependence at 2.5 K by using Equation (1) (Figure 4 c), then Equation (5) describes well the temperature dependence above 2.5 K, but shows increasing deviations at lower temperature. This indicates that the bottleneck process has higher impact at low temperatures, when the number of excited phonons is not sufficient for efficient energy transfer. Note that the direct and Arrhenius processes in Dy_2_LuN@C_80_ have similar rates at 0.2 T, and hence in‐field relaxation rates are around twice as fast up to 3 K, when the Raman process starts to dominate, and the field dependence eventually vanishes by 5 K.

In striking contrast to Dy_2_LuN@C_80_, the relaxation times of Dy_2_ScN@C_80_ at 2.5 K do not depend on the external magnetic field until it exceeds 0.4 T (Figure 4 c). The relaxation times of Dy_2_ScN@C_80_ measured at different temperatures in a field of 0.2 T almost coincide with the zero‐field values and start to show small deviations only below 2 K. This shows that the direct mechanism contributes to the spin relaxation in Dy_2_ScN@C_80_ at considerably higher fields and lower temperatures than in Dy_2_LuN@C_80_, which explains why the coercive field in the magnetic hysteresis of Dy_2_ScN@C_80_ is larger than in Dy_2_LuN@C_80_ (Figure 3 b).

The temperature dependencies of the relaxation times of DyLu_2_N@C_80_ and Dy_2_LuN@C_80_ measured in zero field and in a field of 0.2 T are compared in Figure 4 d. Once the QTM in DyLu_2_N@C_80_ is quenched by the application of a finite field, its relaxation rate is much slower than in Dy_2_LuN@C_80_, and at 2 K the difference between mono‐ and di‐dysprosium NCFs exceeds two orders of magnitude. Intramolecular interactions between dysprosium spins in Dy_2_MN@C_80_ block zero‐field QTM and create a manifold of new low‐energy coupled spin states. Apparently, spin relaxation in Dy_2_LuN@C_80_ at low temperature proceeds via such coupled states and is therefore much faster than in DyLu_2_N@C_80_, which has only single‐ion excited spin states. A similar difference in the low‐temperature relaxation mechanisms was also observed for DySc_2_N@C_80_ and Dy_2_ScN@C_80_.3a, 6b If indeed this is the case, the *U* ^eff^ of the Arrhenius process in Dy_2_MN@C_80_ may be related to the energy difference between the ground and the first excited state of the coupled spin system. At higher temperature, the relaxation rates of DyM_2_N@C_80_ and Dy_2_MN@C_80_ tend to be more similar, which indicates that relaxation via single‐ion states becomes equally efficient for both types of NCFs.

### Single‐ion anisotropy of dysprosium ions in DyM_2_N@C_80_ and Dy_2_MN@C_80_ (M=Sc, Lu)

The central nitride ion is the main source of the magnetic anisotropy in lanthanide NCFs, and the LF is expected to become stronger with decreasing distance between N^3−^ and Dy^3+^. DFT calculations at the PBE level with the 4f‐in‐core effective potential showed that the increase in the ionic radius from Sc^3+^ to Lu^3+^ shortens the Dy−N bond from 2.156 Å in DySc_2_N@C_80_ to 2.090 Å in DyLu_2_N@C_80_.8c Likewise, the DFT‐optimized Dy−N bonds in Dy_2_LuN@C_80_ (2.073 and 2.074 Å) are shorter than those in Dy_2_ScN@C_80_ (2.105 and 2.108 Å). These geometrical changes may substantially affect the LF acting on the dysprosium ions.

**Figure 5:**
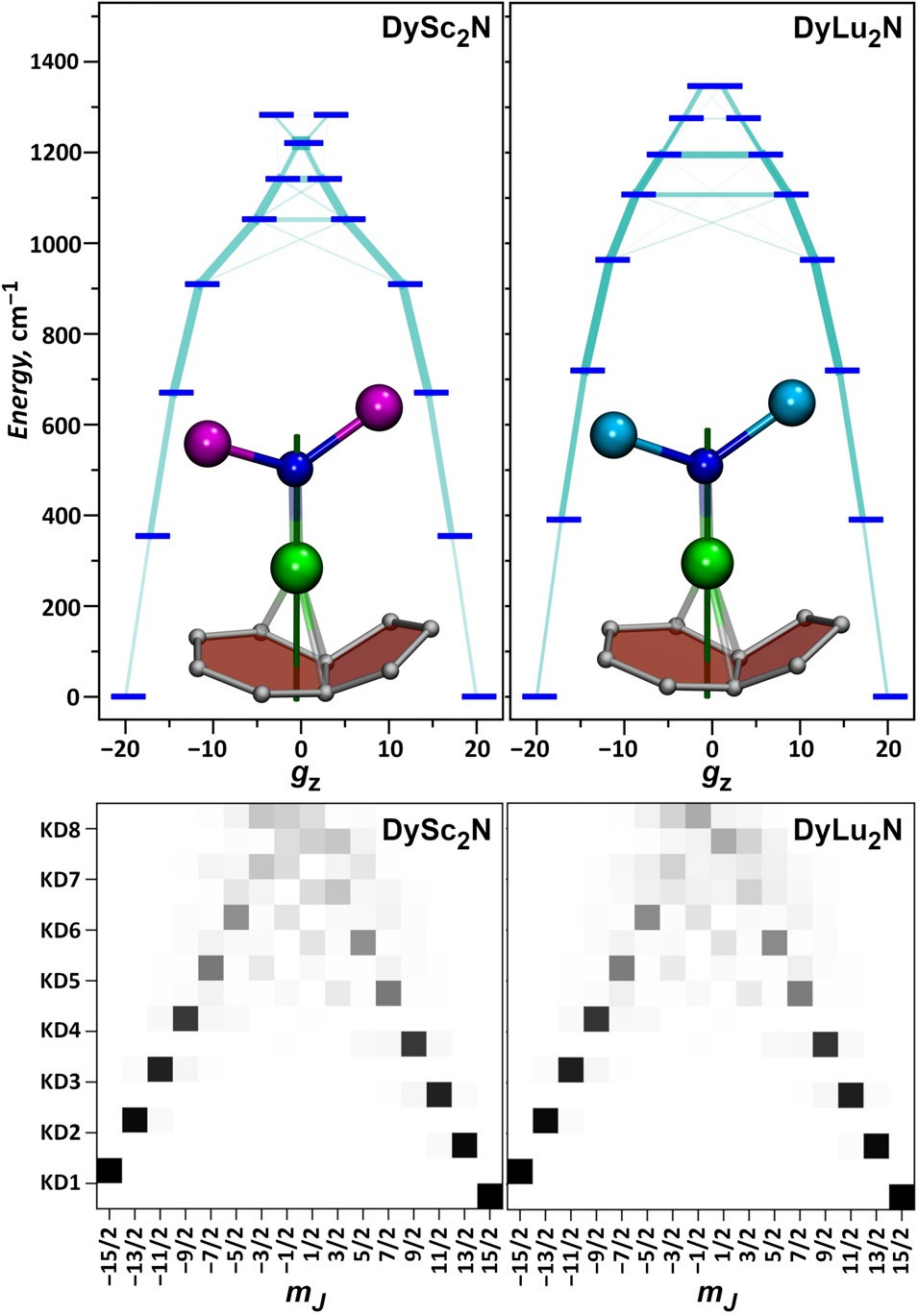
Top: Ab initio computed LF splitting for Dy^3+^ in DySc_2_N@C_80_ and DyLu_2_N@C_80_ molecules. The light‐blue lines visualize the transition probabilities between the KD states. Also shown are DyM_2_N clusters (Dy, green; Sc, magenta; N, blue; Lu, cyan) with the Dy‐coordinated fragment of the fullerene cage (Dy−C distances shorter than 2.4 Å are visualized as bonds). The quantization axis determined by ab initio calculations is shown as a dark‐green line. Bottom: Visualization of the KD wave‐function composition in the $\left|15/2,m_{J}\right.\rangle$ basis (see Table S7 in the Supporting Information for numerical data).

To explore the influence of this effect, we performed ab initio CASSCF/RASSI calculations on the LF splitting in DyLu_2_N@C_80_ and Dy_2_LuN@C_80_ molecules and compared the results with those for the dysprosium‐scandium analogues. For Dy_2_MN@C_80_ molecules, only one dysprosium ion was treated ab initio at a time, and the other dysprosium was replaced by yttrium. The energies of the Kramers doublets (KDs) in DyM_2_N@C_80_ molecules, the pseudo‐spin *g*‐tensor of the ground state KD, and the transition probability in the first KD are presented in Figure 5, Table 2, and Table S7 in the Supporting Information. The calculations show that the Dy^3+^ ions in DyLu_2_N@C_80_ and Dy_2_LuN@C_80_ exhibit high magnetic anisotropy with overall LF splitting of 1340–1360 cm^−1^. The quantization axis is aligned along the Dy−N bond, and the ground Kramers doublet is described as an essentially pure state with |*m_J_*|=15/2. The energy of the second Kramers doublet is predicted to be close to 400 cm^−1^, which ensures that the low‐temperature magnetic properties of both DyLu_2_N@C_80_ and Dy_2_LuN@C_80_ are determined solely by the ground state of Dy^3+^, as in the previously studied dysprosium‐scandium NCFs.2d, 3a, 6b, 6d A comparison of DySc_2_N@C_80_ and DyLu_2_N@C_80_ shows that the increase in metal size from scandium to lutetium leads to an increase in LF splitting from 1284 cm^−1^ (DySc_2_N) to 1348 cm^−1^ (DyLu_2_N). The energy of the second KD (relative to the first KD) also shows an increase from 356 cm^−1^ in DySc_2_N@C_80_ to 391 cm^−1^ in DyLu_2_N@C_80_. As the Dy−N bonds in Dy_2_LuN@C_80_ are somewhat shorter than that in DyLu_2_N@C_80_, the LF splitting for the dysprosium ion in the former is also slightly higher. Likewise, the LF splitting in Dy_2_LuN@C_80_ is somewhat higher than in Dy_2_ScN@C_80_, which also correlates with the shorter Dy−N bond lengths (see Tables S8 and S9).

**Table 2:** Ligand‐field splitting of the Dy^3+^ states and the ground‐state *g*‐tensor in DyM_2_N@C_80_ and Dy_2_MN@C_80_ (M=Sc, Lu) molecules as predicted by ab initio calculations.

	DySc_2_N	DyLu_2_N	Dy_2_ScN		Dy_2_LuN	
			Dy1	Dy2	Dy1	Dy2
KD1 [cm^−1^]	0	0	0	0	0	0
KD2 [cm^−1^]	356	391	399	378	408	403
KD3 [cm^−1^]	666	715	704	698	726	726
KD4 [cm^−1^]	906	960	938	945	967	971
KD5 [cm^−1^]	1050	1104	1081	1084	1109	1112
KD6 [cm^−1^]	1140	1194	1176	1174	1203	1202
KD7 [cm^−1^]	1222	1276	1253	1263	1283	1286
KD8 [cm^−1^]	1284	1348	1336	1333	1365	1366
						
*g_x_*	0.00005	0.00006	0.00006	0.00004	0.00007	0.00007
*g_y_*	0.00006	0.00007	0.00008	0.00006	0.00008	0.00008
*g_z_*	19.83868	19.83507	19.87022	19.85862	19.82302	19.85935
*β* ^[a]^ [°]	1.6	1.8	2.2	2.0	2.2	2.1
KD1↔KD1′^[b]^ [*μ*_B_^2^]	1.1×10^−9^	1.7×10^−9^	2.5×10^−9^	8.0×10^−10^	2.4×10^−9^	2.4×10^−9^
						
*d*(Dy−N)^[c]^ [Å]	2.156	2.090	2.105	2.108	2.074	2.073

[a] *β* is the angle between the Dy−N bonds and the quantization axis of the Dy ions. [b] KD1↔KD1′ denotes the transition probability between the two degenerate states in the first KD, that is, the probability of QTM. [c] The Dy−N bond lengths correspond to the DFT‐optimized structures, see ref. 8c for a comparison of the experimental and computed Dy−N distances.

For the relaxation of magnetization, not only the energies of the KD states, but also the transition probabilities between them as well as the composition of the wave functions in the $\left|J,m_{J}\right.\rangle$ basis are very important (Figure 5, see also Table S7 in the Supporting Information). The first KD with *g_z_* close to 19.8 and infinitesimally small *g_x_* and *g_y_* values has more than 99 % contribution from the ${|m}_{J}|$ =15/2 function for all the discussed NCFs. The probability of a QTM transition within the first KD is only 1.1×10^−9^ *μ*_B_^2^ in DySc_2_N@C_80_ and 1.7×10^−9^ *μ*_B_^2^ in DyLu_2_N@C_80_. The QTM transition probabilities within one KD remain low up to the fourth KD (Figure 5), and similar values are also found for Dy_2_MN@C_80_ molecules. Likewise, transitions between the states of different $m_{J}$ and opposite spin are also not efficient until KD4. The reasons for this situation are rooted in the composition of the KD wave functions, which can be described as essentially pure *m_J_* states up to KD4–KD5 (Figure 5). Thus, ab initio calculations predict that the relaxation of magnetization in all dysprosium NCFs should proceed via the KD5, as indeed was observed experimentally in Dy_2_ScN@C_80_.2d There is no considerable difference between dysprosium‐scandium and dysprosium‐lutetium nitride clusterfullerenes in this regard.

To summarize, the replacement of scandium by lutetium in mixed‐metal nitride clusterfullerenes shortens the Dy−N bonds and increases the LF splitting by 5–10 %. Otherwise, there is no significant difference in terms of KD composition and the expected relaxation pathways via excited KDs.

### Intramolecular interactions of dysprosium magnetic moments in Dy_2_LuN@C_80_

The system of two weakly interacting dysprosium centers with magnetic moments ${\hat{J}}_{1,2}$ can be described by the effective spin Hamiltonian given by Equation (6)

(6) $${\hat{H}}_{spin}={\hat{H}}_{{LF}_{1}}+{\hat{H}}_{{LF}_{2}}-2j_{1,2}{\hat{J}}_{1}{\hat{J}}_{2}$$

in which ${\hat{H}}_{{LF}_{i}}$ is the single‐ion LF Hamiltonian for the *i*th dysprosium site, dysprosium moments ${\hat{J}}_{i}$ are treated in the $\left|J,m_{J}\right.\rangle$ basis, and $j_{1,2}$ is the coupling constant between the localized dysprosium moments. Here, $j_{1,2}$ is treated isotropically in the spirit of the Lines model^18^ and includes both exchange and dipolar interactions.

**Figure 6:**
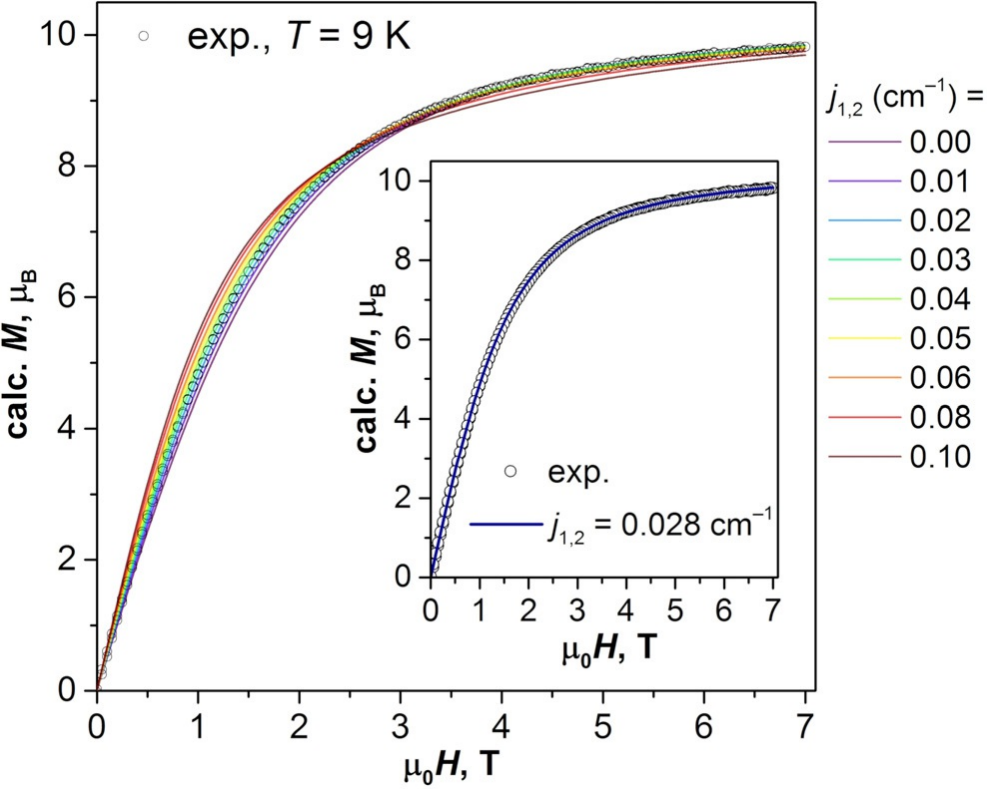
Experimental magnetization curve of Dy_2_LuN@C_80_ measured at 9 K (dotted line) compared with the curves simulated using Equation (5) with different values of *j*_1,2_ from 0 to 0.1 cm^−1^. The inset compares the experimental curve with the simulated one for *j*_1,2_=0.028 cm^−1^.

To determine the $j_{1,2}$ constant for Dy_2_LuN@C_80_, we simulated magnetization curves with different values of $j_{1,2}$ and compared them with the experimental data. In these simulations the angle, *α*, between the single‐ion anisotropy axes of the dysprosium ions is set to 61.7°, as determined by ab initio calculations. The best agreement is achieved for $j_{1,2}$ =0.02–0.03 cm^−1^ (Figure 6). As follows from Equation (6), for the two dysprosium spins oriented at an angle of *α*=61.7°, the energy difference between the states with ferromagnetic (FM) and antiferromagnetic (AF) coupling of the dysprosium ions (*J=*15/2) can be calculated from Equation (7)

(7) $$\Delta E_{FM-AF}=4j_{1,2}J^{2}cos\left(\alpha \right)=225j_{1,2}cos\left(\alpha \right)$$

which gives an estimation of 2.1–3.2 cm^−1^ (3.1–4.6 K) for Dy_2_LuN@C_80_. This energy difference is close to the *U* ^eff^ value of 4.3 K determined for the low‐temperature relaxation process with Arrhenius behavior (see above). The assumption that the relaxation proceeds by excitation to the antiferromagnetically coupled state (i.e., $\Delta E_{FM-AF}$ =*U* ^eff^)2e, 3a allows a more precise estimation of $j_{1,2}$ =0.028±0.001 cm^−1^. This value also gives a reasonable agreement between the experimental and simulated *χT* curves (see Figure S8 in the Supporting Information).

Magnetic Dy⋅⋅⋅Dy interactions have two components, dipolar and exchange. The energy difference between the dipolar interactions in the FM and AF states, $\Delta E_{FM-AF}^{dip}$ , can be calculated by using the well‐known formula for the energy of dipolar interactions between two magnetic moments [Eq. (8)],

(8) $$E_{1,2}^{dip}=-\frac{\mu_{0}}{4\pi R_{1,2}^{3}}\left[3\left(\vec{n_{r}},\vec{\mu_{1}}\right)\left(\vec{n_{r}},\vec{\mu_{2}}\right)-\left(\vec{\mu_{1}},\vec{\mu_{2}}\right)\right]$$

in which $\vec{n_{r}}$ is the normal of the radius vector connecting the two magnetic moments $\vec{\mu_{1}}$ and $\vec{\mu_{2}}$ , *R*_12_ is the distance between them, and *μ*_0_ is the vacuum permeability. For Dy_2_LuN@C_80_ with DFT‐optimized coordinates and $\left|\vec{\mu_{1,2}}\right|$ =10 *μ*_B_, Equation (8) gives $\Delta E_{FM-AF}^{dip}$ =4.8 K. Surprisingly, it appears that the Dy⋅⋅⋅Dy interactions in Dy_2_LuN@C_80_ are solely of dipolar nature, with the exchange term vanishing almost completely. For Dy_2_ScN@C_80_, Equation (8) gives a very similar value of $\Delta E_{FM-AF}^{dip}$ =4.7 K, but as the $\Delta E_{FM-AF}$ energy in Dy_2_ScN@C_80_ is twice as large as in Dy_2_LuN@C_80_, one can conclude that the exchange term for Dy_2_ScN@C_80_ is of a similar size to the dipolar term (Table 3). Thus, substitution of scandium by lutetium in the Dy_2_MN cluster results in considerable variation of the coupling constant, mainly because of the negligible exchange interactions in Dy_2_LuN@C_80_, which also leads to a smaller energy difference between the ferromagnetically and antiferromagnetically coupled states, and through this difference has a strong influence on the relaxation of magnetization at low temperature.

**Table 3:** Exchange and dipolar coupling parameters for di‐Dy EMFs.

	$\Delta E_{FM-AF}$ ^[a]^ [K]	$\Delta E_{FM-AF}^{dip}\;$ [K]	$j_{1,2}\;$ [cm^−1^]	*α* ^[b]^ [°]	*R*_12_^[c]^ [Å]
Dy_2_LuN@C_80_	4.3±0.2	4.8	0.028	61.7	3.559
Dy_2_ScN@C_80_	8.0±0.1^[d]^	4.7	0.055	63.4	3.576

[a] $\Delta E_{FM-AF}$ is considered to be equal to *U* ^eff^ of the low‐temperature Arrhenius process. [b] *α* is the angle between the quantization axes of two Dy ions, determined from ab initio calculations. [c] *R*_12_ is the distance between two Dy ions in DFT‐optimized molecules. [d] The more precise measurements in this work give a somewhat different value from our previous report in ref. 2d and are closer to the data reported earlier in ref. 3a.

### Low‐frequency molecular and lattice vibrations in dysprosium‐metal NCFs

As follows from the ab initio calculations discussed above, dysprosium‐scandium and dysprosium‐lutetium NCFs have very similar single‐ion magnetic anisotropy and ground‐state properties. In addition, the LF splitting in these NCFs is very large and is not relevant for the low‐temperature relaxation of magnetization. Therefore, the difference in their relaxation behavior cannot be explained by the LF splitting and it is necessary to take into account other factors. In addition, the observation of Arrhenius behavior in DySc_2_N@C_80_ and DyLu_2_N@C_80_ with a *U* ^eff^ of 24 K and unconventional parameters also cannot be explained by LF splitting. A deeper insight into the spin–phonon interactions may be needed to explain these phenomena.

The drawbacks of the commonly applied phenomenological approaches to spin–phonon interactions developed in the 1960s are that they are based on the Debye model for vibrations in the crystal and do not provide a clear connection with the microscopic parameters of the molecules (except for the LF splitting, when the Orbach process is involved). For instance, although the relaxation times measured in this work can be well fitted by Equations (1)–(3) and their combinations, the fitted parameters do not provide sufficient insight into the relation between molecular structure and the relaxation of magnetization.

**Figure 7:**
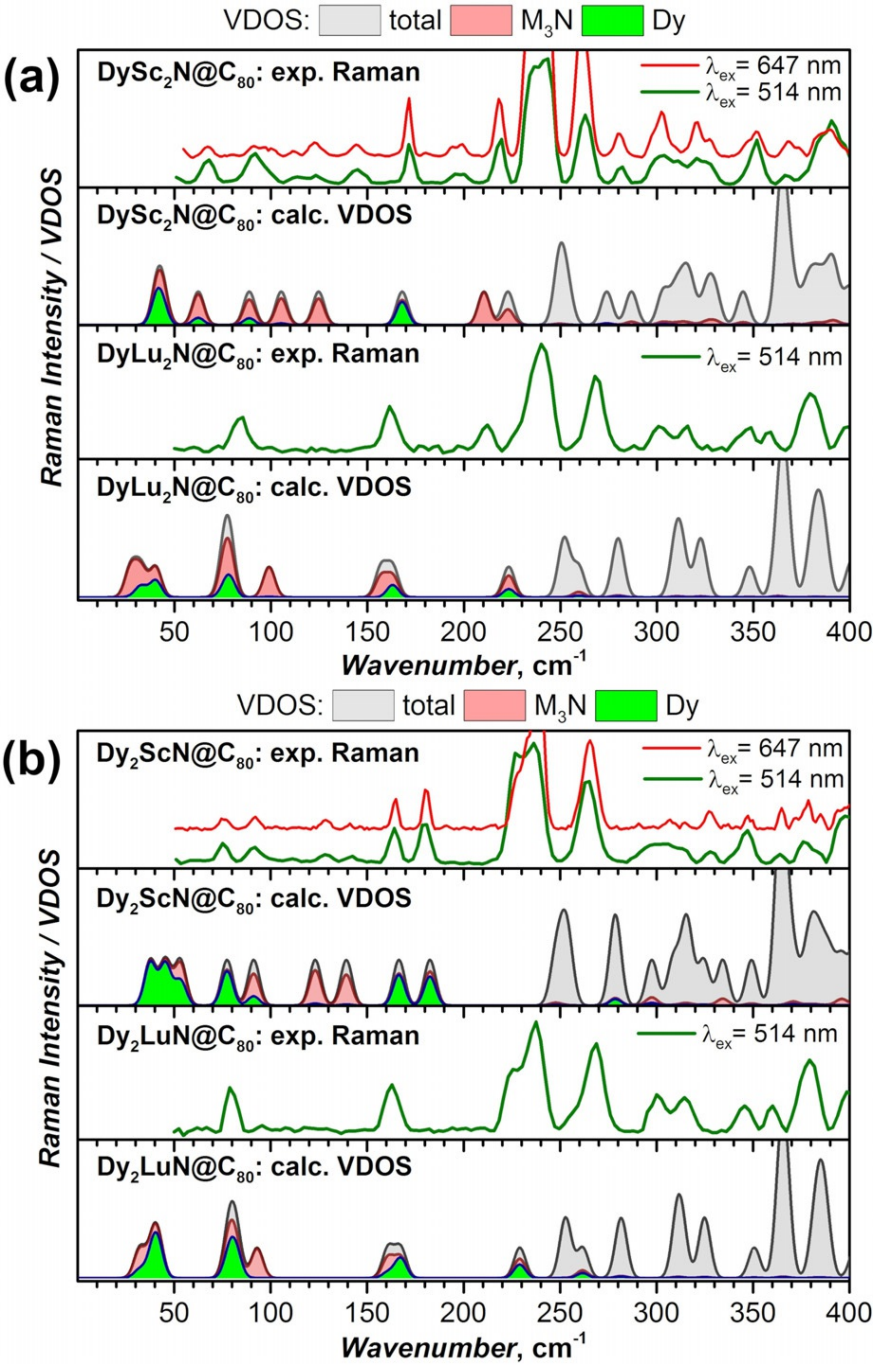
Raman spectra (*T=*77 K, *λ*_ex_=514 or 647 nm) and DFT‐calculated VDOS for (a) DyM_2_N@C_80_ and (b) Dy_2_MN@C_80_ (M=Sc, Lu). The total VDOS is shown in gray, and the contributions to the VDOS of the whole M_3_N cluster and of the Dy atoms are shown in pink and green, respectively.

Recently, Lunghi et al.^17^ analyzed spin–lattice relaxation in molecular magnets by using quantum spin dynamics, considering explicit vibrations of the molecule and deriving the spin–phonon coupling parameters from ab initio calculations. They found that spin–phonon relaxation via anharmonic phonons may also result in Arrhenius behavior, but with the *U* ^eff^ corresponding to half of the vibrational frequency. Furthermore, other research teams analyzed the locality of the spin–phonon interaction and concluded that molecular vibrations spatially localized close to the metal center usually have the strongest contribution to the spin relaxation.^17^, ^19^ These findings show that the Debye model is oversimplified for the analysis of spin–phonon relaxation in molecular magnets^20^ and an analysis of the real vibrational spectra may give better insight into the relaxation mechanism.^21^ With this in mind, we decided to analyze the low‐frequency part of the vibrational spectra of the NCFs. The experimental Raman spectra of DyM_2_N@C_80_ and Dy_2_MN@C_80_ (M=Sc, Lu; limited to frequencies above 50 cm^−1^ due to instrument limitations) are compared in Figure 7 with the vibrational density of states (VDOS), computed by DFT for isolated molecules. In addition to the total VDOS, Figure 7 also shows contributions to the VDOS of the whole metal nitride cluster and of only the dysprosium atoms. The computed and experimental frequencies of the cluster‐based modes are presented in Table 4.

**Table 4:** Experimental and DFT‐computed frequencies of cluster‐based vibrations in DyM_2_N@C_80_ and Dy_2_MN@C_80_ (M=Sc, Lu).^[a]^

Vibrational frequency [cm^−1^]											
DySc_2_N^[b]^			Dy_2_ScN			DyLu_2_N			Dy_2_LuN		
Calcd	Exp		Calcd	Exp		Calcd	Exp		Calcd	Exp	
41		R	38		R_Sc‐N_	27		R_Dy‐N_	32		R
44		R	45		R	33		R	39		R
62	67	R_Dy‐N_	53		R	40		R	42		R
89	91	T+δ_DyNSc_	77	75	δ_DyNDy_	75	84	T+δ_LuNLu_	78	79	δ_DyNLu_
105	111	T+δ_DyNSc_	91	91	T	78	84	T+δ_LuNLu_	80	79	T+δ_DyNDy_
125	122	δ_ScNSc_	123	129	δ_DyNSc_	79	84	δ_DyNLu_	82	79	T+δ_DyNDy_
168	172	T+ν_Dy‐N_	139	142	γ_N_	99		γ_N_	93		γ_N_
210	198	γ_N_	167	164	T+δ_DyNDy_	157	162	T+ν_Lu‐N_	161	163	T+ν_Lu‐N_
223	218	T+δ_DyNSc_	183	180	T+ν_Dy‐N_	163	162	T+ν_Dy‐N_	167	163	T+ν_Dy‐N_
						223	212	ν_M‐N_	229		ν_M‐N_

[a] Designation of cluster modes: R, rotation (libration); T, translation; δ_MNM′_, M−N−M′ bending; γ_N_, nitrogen out‐of‐plane displacement; ν_M‐N_, M−N stretching vibration. [b] Experimental Raman spectrum of DySc_2_N@C_80_ has a peak at 145 cm^−1^ that cannot be assigned on the basis of calculation results.

EMFs have rather peculiar vibrational spectra as their molecules consist of two semi‐independent units, the vibrations of which show almost no overlap in the energy scale, as can be well seen in Figure 7. Thus, the vibrations of the relatively rigid carbon cage occur at frequencies exceeding 240 cm^−1^, whereas the frequencies of metal‐involving modes rarely exceed 230 cm^−1^, because the metal atoms are much heavier than carbon atoms. Only in the border range of 220–260 cm^−1^ do the squashing cage modes partially mix with the “breathing” mode of the nitride cluster, in which all three metal atoms move radially in one phase along the M−N bonds. Other metal‐based vibrations occur at frequencies below 200 cm^−1^. When the nitride cluster is encapsulated inside the fullerene, its external degrees of freedom (i.e., translations and rotations) are transformed into internal ones (i.e., molecular vibrations). The frustrated rotations (i.e., librations) are the lowest‐frequency intramolecular modes predicted to be close to 30–40 cm^−1^ in dysprosium‐lutetium NCFs and at 40–60 cm^−1^ in dysprosium‐scandium NCFs. The frustrated translations are mixed with deformations of the cluster (such as in‐plane oscillations of the M−N−M angles). In DyLu_2_N@C_80_ and Dy_2_LuN@C_80_, these modes are clustered into two groups close to 80 and 160 cm^−1^ (because dysprosium and lutetium have similar atomic masses, both dysprosium‐lutetium NCFs have very similar VDOS). In dysprosium‐scandium NCFs, such modes are more uniformly spread in the 80–200 cm^−1^ range. Finally, the nitrogen out‐of‐plane mode also falls in the range of 90–210 cm^−1^.

The results of the computations agree reasonably well with the experimental Raman spectra. Above 220 cm^−1^, the spectra of all the NCFs are quasi‐continuous because of densely spaced cage vibrations. The calculations seem to overestimate the cage frequencies by around 10 %. In the cluster frequency range, DyLu_2_N@C_80_ and Dy_2_LuN@C_80_ exhibit only two Raman peaks, at around 80 and 162 cm^−1^, close to the predicted frequencies of the mixed translation/deformation modes. The dysprosium‐scandium NCFs exhibit richer spectral patterns, and most of the observed peaks can be reliably assigned to the computed modes, as listed in Table 4 (see refs. 22 for a more detailed discussion of the vibrational spectra of MSc_2_N@C_80_ NCFs). Due to technical limitations, we cannot record the spectra below 50 cm^−1^, which precludes experimental observation of the cluster librations in dysprosium‐lutetium NCFs. However, good agreement between experiment and theory for the cluster modes above 50 cm^−1^ ensures that the calculated frequencies are not far from reality. In addition, in some of the earlier Raman studies of the NCFs, peaks at around 30–40 cm^−1^ were reported for Dy_3_N@C_80_, Lu_3_N@C_80_, and some other M_3_N@C_80_ molecules.8a, 23

### Librations of the cluster and lattice phonons in spin relaxation

The low‐frequency vibrations localized on the metal nitride cluster of the M_3_N@C_80_ molecules are expected to mediate energy transfer between the spin and a thermal bath. The rotational motions of the cluster are especially of interest from the point of view of low‐temperature spin relaxation because they not only happen at low frequencies (i.e., in the relevant energy range), but also because they may help to conserve the total angular momentum when the spin flips. The relevance of the Einstein–de Haas effect on the single‐molecule level was demonstrated by Wernsdorfer and co‐workers for TbPc_2_ (Pc=phthalocyanine) grafted on a carbon nanotube (CNT).^24^ To conserve the total momentum, the spin reversal of terbium had to result in rotation of the TbPc_2_ molecule around the terbium quantization axis. However, because the molecule was rigidly bonded to the CNT, to fulfill the rotational invariance, the rotational momentum had to be transferred to the momentum of the phonon propagating along the nanotube. As a result, the efficient spin reversal proceeded by the direct mechanism when the external magnetic field created a Zeeman splitting matching the frequency of the longitudinal nanotube phonon. In a dysprosium nitride cluster, the reversal of the spin aligned along the Dy−N bond should induce rotation of the cluster around this bond. But as discussed above, interaction with the fullerene cage restricts the rotational motion of the cluster and turns it into a vibration, albeit retaining its rotational character. In DyLu_2_N@C_80_ and DySc_2_N@C_80_, the frequencies of the corresponding cluster librations are predicted at 27 and 62 cm^−1^, respectively (Table 4). However, localized molecule vibrations at the Γ point do not transfer moment either, and hence their dispersions and interactions with lattice phonons should be studied further.

Consideration of the lattice phonons in EMF solids is also necessary because their frequency range is likely to overlap with low‐frequency intramolecular vibrations. Unfortunately, experimental information on lattice phonons in EMFs is very limited. To the best of our knowledge, there has been only one study of monometallofullerenes by inelastic neutron scattering, which showed almost featureless VDOS in the low‐frequency range.^25^ Far‐IR^25^, ^26^ as well as the aforementioned Raman studies proved the presence of some low‐frequency modes in EMFs, but metal‐based intramolecular vibrations could not be distinguished from the lattice modes, and, in addition, the selection rules limit the optical activity only to the Γ point. Lattice vibrations of empty fullerenes, and especially C_60_, are much better studied. According to inelastic neutron scattering and ab initio computations, the lattice phonons of C_60_ exhibit rather strong dispersion and cover the range up to 60–70 cm^−1^,^27^ whereas optical spectroscopic studies revealed the bands of librational modes at 7 and 18 cm^−1^, and those of translational modes at 28, 41, and 59 cm^−1^.^28^ Thus, we can tentatively suggest that the frequency ranges of lattice modes and the intramolecular cluster vibrations of EMFs do overlap, which may lead to significant mode mixing.

In the absence of experimental information, we performed computational modeling of the lattice phonons of M_3_N@C_80_ to analyze their possible interaction with intramolecular vibrations. Complete calculations of the phonons of fullerene crystals at the DFT level are hardly feasible at this moment, and therefore simulations were performed by using the less demanding density‐functional based tight‐binding (DFTB) approach.^29^ The pair‐atomic interaction potentials available for Sc_3_N@C_80_ showed a reasonable prediction of the molecular geometry and vibrations.^30^ As potentials for other lanthanide atoms of interest are not known, we used the Sc_3_N@C_80_ model to determine the Hessian and then computed the dispersion spectra and vibrational eigenvectors for different lanthanide‐containing NCFs by using a proper mass correction in a dynamic matrix.

**Figure 8:**
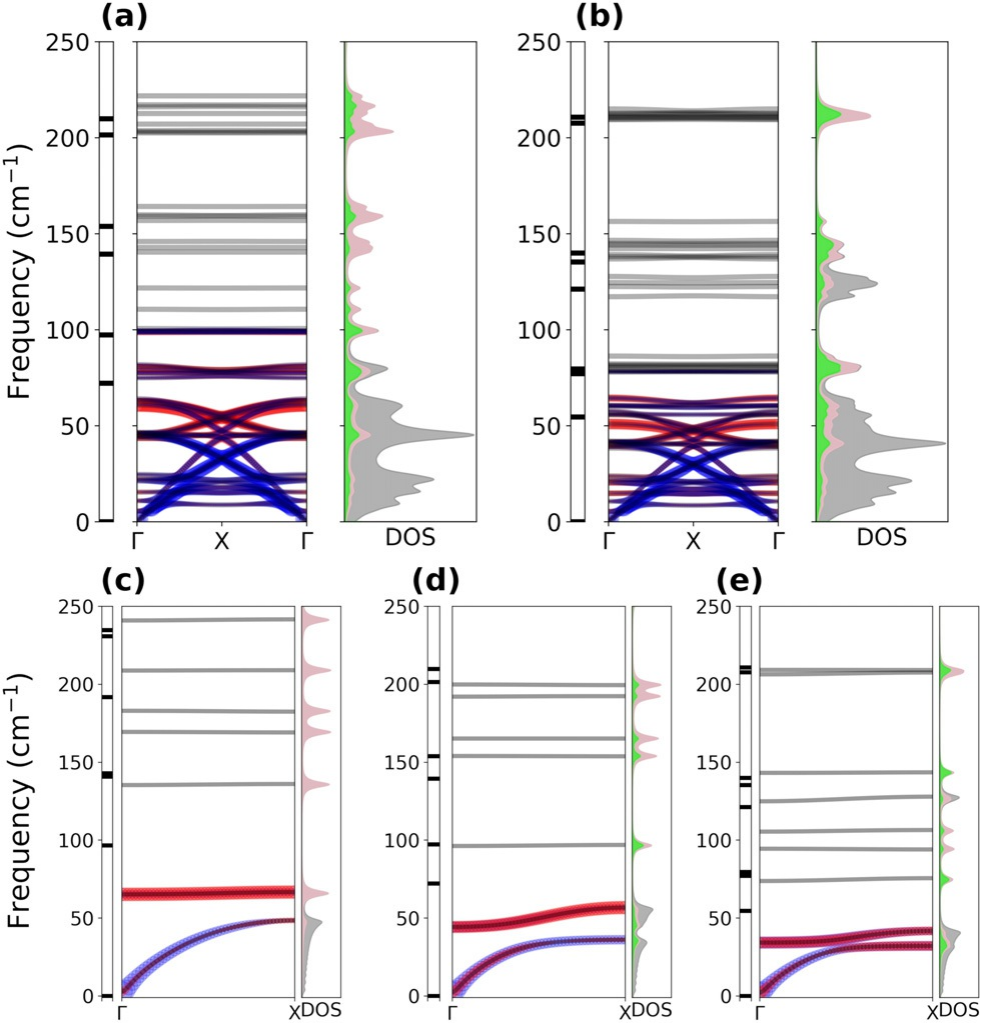
Phonon spectra for five model systems: 3D crystal with FCC packing of (a) DySc_2_N@C_80_ and (b) DyLu_2_N@C_80_; 1D chains of (c) Sc_3_N@C_80_, (d) DySc_2_N@C_80_, and (e) DyLu_2_N@C_80_. For each system, the right panel represents the frequencies computed for isolated molecules, the central panel shows phonon dispersions along the Γ‐X high‐symmetry path with information on band compositions (see text for details), and the left panel demonstrates the projected VDOS (total, gray; M_3_N cluster, pink; Dy contribution, green).

The model lattice of M_3_N@C_80_ molecules was simulated with face‐centered cubic (FCC) packing with the optimized unit cell parameter *a* of 15.5 Å and the distance between the centers of fullerene molecules of 11 Å (see Figure S11 in the Supporting Information). The vibrational spectra computed for isolated DySc_2_N@C_80_ and DyLu_2_N@C_80_ molecules are compared in Figure 8 with those of the crystal phase, and dispersion of the phonons along the high‐symmetry line Γ‐X can be seen. In both cases, the DFTB‐computed spectra of the isolated molecules start above 50 cm^−1^, which is just on the borderline for the acoustic bands of the crystals. The molecular modes away from the frequency range of the acoustic modes show noticeable Davydov splitting but negligible *k*‐dispersion. But the local modes close to the acoustic bands intertwine with the dispersed lattice bands giving rise to a dense phonon structure starting from zero frequency on. Three clear acoustic modes are perturbed by a set of what appear to be local modes with fluid character across *k*‐space. To follow the possible mode mixing in *k*‐space, we chose Γ‐point vibrational eigenvectors as a basis space, in which eigenvectors computed at different *k* values were projected.

The results of this projection analysis for one pure acoustic mode and for one with cluster libration character are presented in Figure 8 a,b using a color code (blue for the acoustic and red for the libration) and a scatter plot, for which the size of the dots is proportional to the magnitude of the projection (Figure 8). In this representation we can see and quantify how strongly the acoustic mode couples to more localized modes as a function of *k*. This redistribution is also reflected in the projected DOS in Figure 8, with the acoustic band showing a steady increase of cluster contributions as the energy increases. This model computation clearly shows that substantial mode mixing indeed takes place in *k*‐space. However, the 3D model is challenging to grasp due to extensive mixing and a high density of states. For illustrative purposes, we simplified the model to one dimension and considered a linear chain of M_3_N@C_80_ molecules with *a=*10.75 Å (see Figure S11 in the Supporting Information).

Figure 8 c–e shows dispersion relationships computed for 1D chains of three NCF molecules, namely Sc_3_N@C_80_, DySc_2_N@C_80_, and DyLu_2_N@C_80_. The spectra contain only one prominent acoustic band with a large dispersion of 50 cm^−1^ and two bands with a smaller dispersion of 10 cm^−1^ produced by on‐site rotational degrees of freedom. In 1D Sc_3_N@C_80_, the lattice and cluster modes are high in frequency, the cluster libration band is flat, and a weak mixing with the acoustic mode can be detected only around the X symmetry point. With the increase in mass in DySc_2_N@C_80_, the cluster‐based frequencies decrease faster than those of the lattice phonons, and the degree of mixing increases as manifested in the considerable dispersion of the intramolecular mode propelled by the acoustic band. Furthermore, the local and acoustic mode frequencies are even closer in 1D DyLu_2_N@C_80_, and therefore the mode mixing is much more pronounced.

The mixing of cluster libration modes with the lattice phonons outside of the Γ point has profound consequences for momentum transfer. These results show how in the first instance the librations, still being local modes, can assist the total conservation of momentum during spin flip and would be able to redistribute the excess angular momentum onto the lattice at some *k* values. Mixing of the cluster rotations with the lattice phonons thus facilitates spin reversal by interaction of the local and lattice modes in *k*‐space. Based on this conclusion, we tentatively suggest that the Arrhenius behavior with a *U* ^eff^ of 24 K (17 cm^−1^) observed in the temperature dependence of magnetization relaxation times of DySc_2_N@C_80_ and DyLu_2_N@C_80_ (Figure 4 a) may be caused by spin reversal assisted by the rotational modes of the M_3_N cluster. *U* ^eff^ in this case should correspond not to the frequency in the Γ point, but rather to the frequencies in the high density of states of the phonons with strong mixed character. In addition, the difference in the masses of the dysprosium‐scandium and dysprosium‐lutetium nitride clusters will result in a different degree of mixing between the cluster librations and lattice phonons, thus leading to a different efficiency of the spin–lattice relaxation.

## Conclusions

In this work we have studied the magnetic properties of the nitride clusterfullerenes DyLu_2_N@C_80_ and Dy_2_LuN@C_80_ and analyzed how substitution of scandium by lutetium in the mixed‐metal clusterfullerenes DyM_2_N@C_80_ and Dy_2_MN@C_80_ affects the single‐molecule magnetism thereof. DyLu_2_N@C_80_ and Dy_2_LuN@C_80_ have been found to be SMMs with a blocking temperature of 9.5 and 8 K, respectively. DyLu_2_N@C_80_ exhibits a higher blocking temperature, longer relaxation times, and broader hysteresis than the dysprosium‐scandium analogue DySc_2_N@C_80_. Both DySc_2_N@C_80_ and DyLu_2_N@C_80_ feature zero‐field QTM, and when the QTM is quenched in a finite field of 0.2 T, the magnetization relaxation times of both compounds show Arrhenius behavior with an effective barrier of 24 K.

Dy_2_LuN@C_80_ and Dy_2_ScN@C_80_ have identical blocking temperatures, but show different temperature and field dependence of the relaxation times. In particular, a direct relaxation mechanism with enhanced field dependence is observed for Dy_2_LuN@C_80_ below 5 K, whereas the relaxation times of Dy_2_ScN@C_80_ remain independent of field until the field exceeds 0.4 T. The magnetization relaxation times of Dy_2_LuN@C_80_ show Arrhenius behavior with an effective barrier of 4.3 K, which has been assigned to the energy of the excited state with antiferromagnetic coupling of the dysprosium moments, $\Delta E_{FM-AF}$ . In Dy_2_ScN@C_80_, the energy of this state is around two‐fold higher, at 8.0 K. Because Dy_2_LuN@C_80_ and Dy_2_ScN@C_80_ have almost identical energies of intramolecular dipolar interactions of 4.7 K, the considerable difference in their $\Delta E_{FM-AF}$ values is attributed to the strong variation in the exchange coupling when scandium is substituted by lutetium. Essentially, the magnetic moments in Dy_2_LuN@C_80_ show only dipolar interactions and their exchange coupling vanishes.

To aid the understanding of possible spin–phonon energy exchange, the low‐frequency vibrational spectra of the dysprosium‐lutetium and dysprosium‐scandium NCFs were analyzed experimentally and with the help of DFT calculations. Enclosing the M_3_N cluster inside the fullerene cage transforms its rotational degrees of freedom into molecular vibrations, which retain rotational character and are dubbed as librations of the cluster. The low frequencies of these modes lead to overlap with the frequency range of the lattice phonons. Furthermore, projection analysis also revealed the strong mixing of the local cluster librations with acoustic phonons of the fullerene lattice in the *k*‐space away from the Γ point. As a result, these modes are predicted to facilitate the relaxation of magnetization by helping to conserve momentum during the spin reversal. Thus, the results of our study emphasize that the mixing of local and lattice modes in *k*‐space may be an important mechanism of the spin–lattice relaxation and should be considered for other molecular magnets.

## Experimental Section

Powder samples of fullerenes for magnetometry studies were prepared by drop‐casting from toluene or CS_2_ solutions. The magnetic properties were studied with a Quantum Design MPMS3 Vibrating Sample Magnetometer (VSM). Modeling of the magnetization curves and the spin Hamiltonian solution was accomplished with the PHI program.^31^

Raman spectra were recorded at 77 K with a T 64000 triple spectrometer (Jobin Yvon) using an excitation wavelength of *λ*_ex_=514 nm (Ar^+^ laser) or *λ*_ex_=647 nm (Kr^+^ laser). The samples for Raman measurements were drop‐cast onto single‐crystal KBr disks.

DFT calculations on isolated M_3_N@C_80_ molecules were performed at the PBE‐D level with a plane‐wave basis set and the corresponding projector augmented‐wave potentials, treating 4f electrons as a part of the core as implemented in the VASP 5.0 package.^32^ These calculations employed a cubic unit cell with a lattice parameter of 25 Å and the atomic cut‐off energy was set to 400 eV. The precision was set to be “accurate” with real‐space projector operators optimized down to 10^−4^ eV per atom. The Γ‐point Hessian matrix and then the vibration frequencies (or Γ‐point phonons) were determined by using density functional perturbation theory as implemented in VASP.

The ab initio energies and wave functions of LF (ligand field) multiplets for the dysprosium‐lutetium NCF molecules were calculated at the CASSCF/SO‐RASSI level of theory with atomic natural orbital extended relativistic basis set (ANO‐RCC)^33^ of the valence double‐zeta (VDZ) quality using the quantum chemistry package MOLCAS 8.0.^34^ The active space of the CASSCF calculations included the 4f shell, that is, 11 active electrons and 7 active orbitals. All 21 sextet states and 108 quartets and only 100 doublets were included in the state‐averaged CASSCF procedure and further used in the RASSI procedure with a spin–orbit Hamiltonian. The single‐ion magnetic properties and LF parameters were calculated on the basis of the ab initio data with the use of a SINGLE ANISO module.^35^

Phonon spectra modeling and analysis were performed with in‐house Python scripts based on ASE libraries and with the improved version of some functions.^36^ The DFTB+ software29b was used as a force derivation code in the Calculator class of ASE. The finite differences method was used in phonon calculations with atomic displacement of 0.03 Å along each Cartesian axis. Prior to phonon calculations, 1D and 3D systems (see Figure S11 in the Supporting Information) were optimized with a quasi‐Newton algorithm down to 0.0002 eV Å^−1^ with 2 *k*‐point sampling in each periodic direction. In all calculations, the non‐charge‐self‐consistent model was employed with the Slater–Koster parameters developed for similar types of system.^37^

## Conflict of interest

The authors declare no conflict of interest.

## Supporting information

As a service to our authors and readers, this journal provides supporting information supplied by the authors. Such materials are peer reviewed and may be re‐organized for online delivery, but are not copy‐edited or typeset. Technical support issues arising from supporting information (other than missing files) should be addressed to the authors.

Supplementary
