# Supplementary material for: Single‐Molecule Magnets DyM2N@C80 and Dy2MN@C80 (M=Sc, Lu): The Impact of Diamagnetic Metals on Dy3+ Magnetic Anisotropy, Dy⋅⋅⋅Dy Coupling, and Mixing of Molecular and Lattice Vibrations
Source: Chemistry. 2020 Feb 6;26(11):2436–49. doi: 10.1002/chem.201904879 (PMC7065109; doi:10.1002/chem.201904879)
Supplement: Supplementary file 1 — Supplementary [file CHEM-26-2436-s001.pdf]

CHEMISTRY
A European Journal

Supporting Information

Single-Molecule Magnets $DyM_2N@C_{80}$ and $Dy_2MN@C_{80}$ (M = Sc, Lu): The Impact of Diamagnetic Metals on Dy^{3+} Magnetic Anisotropy, Dy···Dy Coupling, and Mixing of Molecular and Lattice Vibrations

Lukas Spree,[a] Christin Schlesier,[a] Aram Kostanyan,[b] Rasmus Westerström,[b, c] Thomas Greber,[b] Bernd Büchner,[a] Stanislav M. Avdoshenko,*[a] and Alexey A. Popov*[a]

chem_201904879_sm_miscellaneous_information.pdf

Single molecule magnetism of $DyM_2N@C_{80}$ and $Dy_2MN@C_{80}$ (M = Sc, Lu): The impact of diamagnetic metals on the magnetic anisotropy of Dy^{3+} ions, Dy···Dy coupling, and the mixing of molecular and lattice vibrations

Lukas Spree,[1] Christin Schlesier,[1] Aram Kostanyan,[2] Rasmus Westerström,[2,3] Thomas Greber,[2] Bernd Büchner,[1] Stanislav M. Avdoshenko,[1]* Alexey A. Popov[1]*

Supporting Information

Relaxation times of $DyLu_2N@C_{80}$ S2

Relaxation times of $Dy_2LuN@C_{80}$ S5

Relaxation times of $Dy_2ScN@C_{80}$ S9

Experimental and calculated χT curves S12

DFT-optimized atomic coordinates S13

Ab initio calculations of LF splitting and wavefunction composition in $DyM_2N@C_{80}$ S21

Ab initio calculations of LF splitting and wavefunction composition in $Dy_2LuN@C_{80}$ S22

Ab initio calculations of LF splitting and wavefunction composition in $Dy_2ScN@C_{80}$ S23

1D and 3D fullerene structures used in phonon calculations S24

Magnetization relaxation times

The sample was first magnetized to saturation, then the field was swept to 0 T with the highest possible sweep rate, and then decay of magnetization of recorded, and the decay curve was then fitted with stretched exponential function:

$$M(t) = M_{eq} + (M_0 - M_{eq}) \exp\left[-\left(\frac{t}{\tau_M}\right)^{\beta}\right] \quad \text{(S1)}$$

Where M_{eq} and M_0 are the equilibrium and initial magnetizations, respectively, τ_M is a characteristic relaxation time and β is an additional parameter that corresponds to the time-dependent decay rate.

Table S1. Relaxation times of $DyLu_2N@C_{80}$ measured in zero magnetic field (fitting with two stretched exponentials)[a]

T, K	*τ₁*, s	±	β₁	±	*M_eq*	±	*M_01*	±	τ₂, s	±	β₂	±
1.8	54.4	0.8	0.69	0.011	2.1E−6	2.5E−7	4.9E−5	2.4E−6	577	156	0.48	0.069
2.0	46.8	0.5	0.61	0.004	−5.2E-7	1.3E−8	5.3E−5	4.3E−7	1806	29	0.49	0.004
2.2	40.5	0.7	0.60	0.005	−3.3E-7	7.5E−9	5.1E−5	5.9E−7	1548	26	0.53	0.004
2.5	36.8	0.5	0.69	0.009	−1.0E-7	1.8E−7	3.7E−5	1.1E−6	542	69	0.42	0.031
2.8	32.8	0.6	0.70	0.009	−5.9E-7	8.9E−8	3.1E−5	8.5E−7	335	37	0.37	0.016
3.0	31.8	0.5	0.71	0.010	−3.2E-7	7.1E−8	2.9E−5	8.8E−7	337	36	0.42	0.019
3.2	30.7	0.5	0.73	0.011	−2.4E-7	4.4E−8	2.7E−5	8.2E−7	274	26	0.44	0.016
3.6	27.9	0.8	0.75	0.019	−9.8E-7	2.0E−8	2.2E−5	1.0E−6	186	19	0.47	0.016
4.0	25.1	1.8	0.77	0.045	−7.8E-8	1.1E−8	1.8E−5	1.8E−6	98	16	0.45	0.019
4.4	29.4	4.7	0.88	0.115	−5.8E-8	9.2E−9	1.1E−5	2.5E−6	50	12	0.42	0.022
5.0	21.2	6.1	0.68	0.118	−1.8E-8	1.2E−8	1.4E−5	7.7E−6	44	35	0.46	0.079

[a] Parameters of the second exponential (with longer time τ_2) are reliable only for 2.0 and 2.2 K as decays were measured long enough. For other temperatures measurement times were not sufficiently log to fully capture longer decay since the main focus was on the short QTM process (τ_1), and τ_2 are likely to be underestimated, especially at the lowest temperatures

Table S2. Relaxation times of $DyLu_2N@C_{80}$ measured in a field of 0.2 T

T, K	τ, s	±	β	±	*M_eq*	±	*M_0*	±
2.0	473144	22120	0.49	0.002	4.5E−5	2.0E−6	1.6E−4	2.1E−6
2.2	133900	4421	0.59	0.002	4.5E−5	1.7E−6	1.5E−4	1.7E−6
2.5	37713	548	0.69	0.001	4.4E−5	7.8E−7	1.5E−4	7.9E−7
2.8	14603	264	0.74	0.002	4.6E−5	9.4E−7	1.5E−4	9.7E−7
3.0	7314	36	0.79	0.001	5.4E−5	2.3E−7	1.5E−4	2.5E−7
3.2	4837	31	0.81	0.001	5.1E−5	3.2E−7	1.5E−4	3.3E−7
3.6	2272	2	0.84	0.000	4.8E−5	4.2E−8	1.4E−4	5.2E−8
4.0	1270	1	0.82	0.001	4.1E−5	4.4E−8	1.4E−4	8.6E−8
4.4	735	1	0.79	0.001	3.6E−5	2.9E−8	1.3E−4	1.3E−7
5.0	345	1	0.80	0.002	3.1E−5	2.7E−8	1.2E−4	1.4E−7
5.5	198	1	0.75	0.002	2.8E−5	1.6E−8	1.1E−4	2.1E−7
6.0	123	1	0.72	0.003	2.5E−5	1.2E−8	9.5E−5	2.7E−7
7.0	58	1	0.69	0.004	2.1E−5	7.2E−9	6.6E−5	2.6E−7

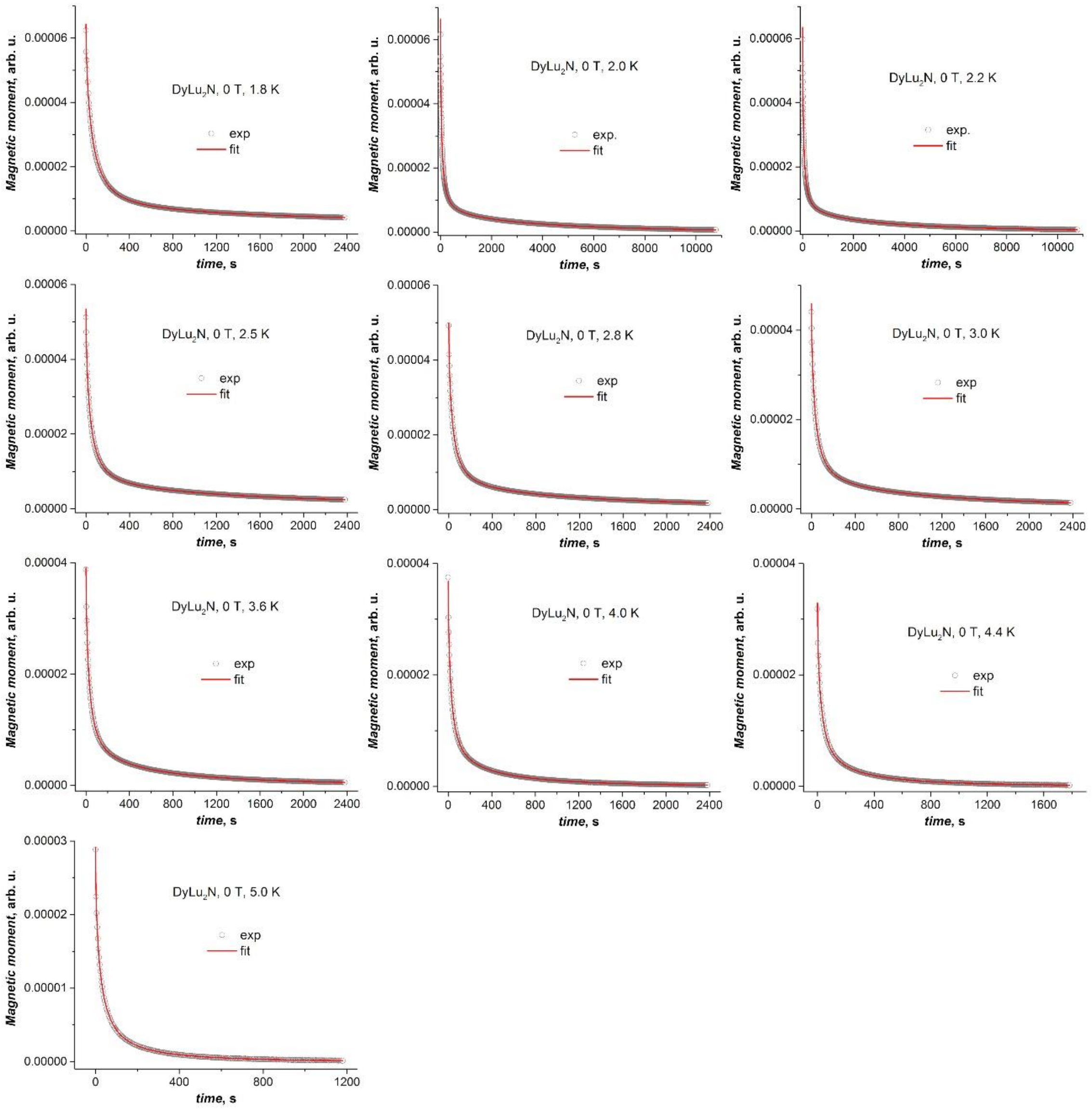

Figure S1. Selected magnetization decay curves of $DyLu_2N@C_{80}$ measured at different temperatures in zero magnetic field. Decay curves show two clear regimes with fast and also relaxation. Fitting of such curves with one stretched exponential function gave unreasonably small β values of less than 0.2. Therefore, curves were fitted with two stretched exponentials, which gave fast and long processes. We assign the fast process to the QTM relaxation of the major part of the sample and discuss only this process in the manuscript. The slow relaxation process may have different reasons, including slow relaxation of the magnet and deviation of the field from zero. The complete set of determined values is listed in Table S1.

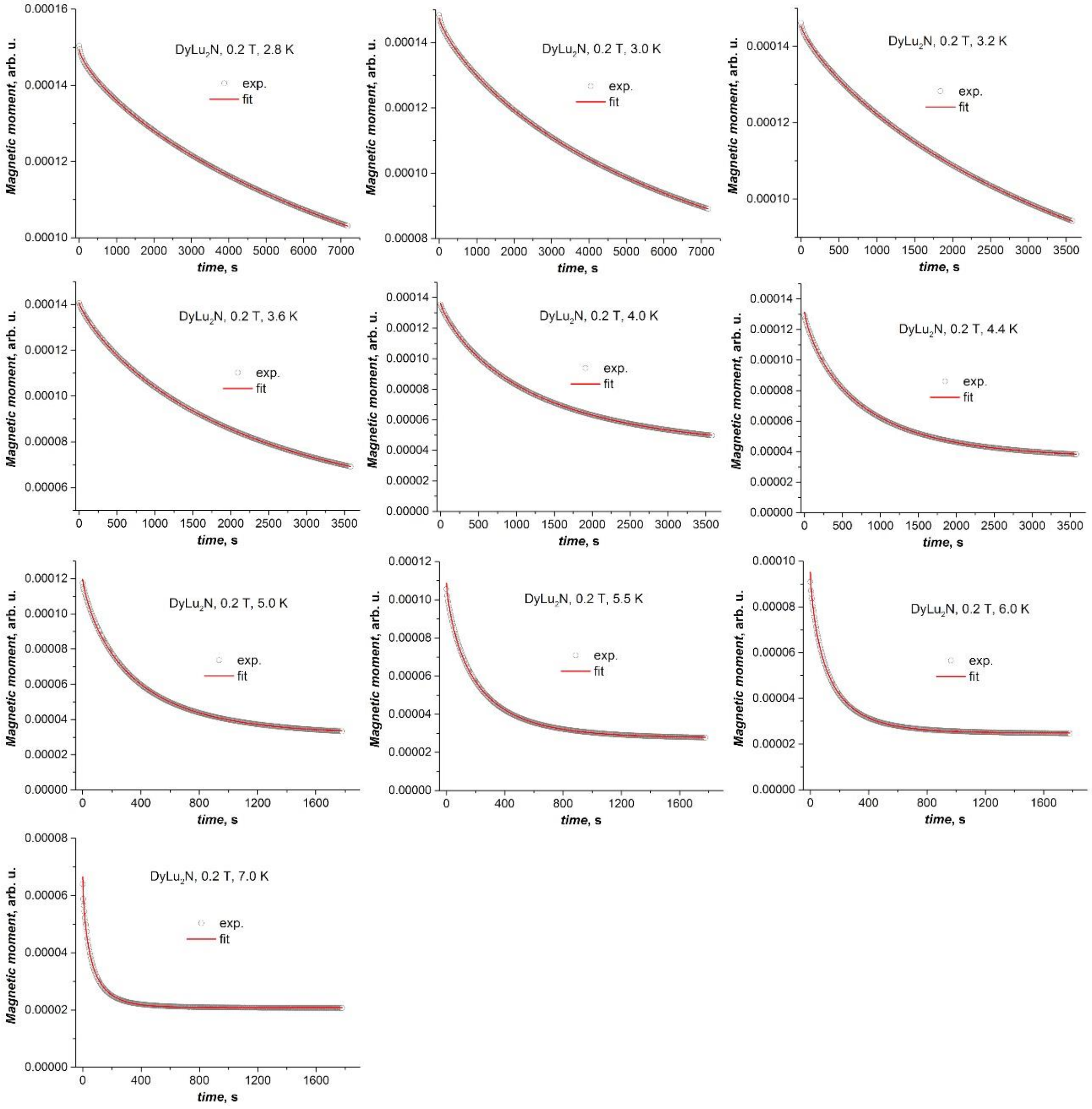

Figure S2. Selected magnetization decay curves of $DyLu_2N@C_{80}$ measured at different temperatures in a field of 0.2 T. The complete set of determined values is listed in Table S2.

Table S3a. Relaxation times of $Dy_2LuN@C_{80}$ measured in zero field

T, K	*τ*, s	±	β	±	M_{eq}	±	M_0	±
1.8	4256	7	0.76	0.001	−5.4E−7	1.3E−8	6.2E−5	8.6E−8
1.9	3651	6	0.76	0.001	−4.8E−7	7.5E−9	6.1E−5	8.1E−8
2.0	3144	4	0.75	0.001	−3.9E−7	1.5E−8	6.1E−5	8.2E−8
2.2	2443	4	0.75	0.001	−4.3E−7	1.0E−8	6.0E−5	7.6E−8
2.35	2058	3	0.75	0.001	−3.7E−7	5.4E−9	6.0E−5	6.9E−8
2.5	1748	2	0.76	0.001	−2.9E−7	5.2E−9	5.9E−5	6.7E−8
2.7	1398	3	0.76	0.001	−2.4E−7	5.2E−9	5.9E−5	9.1E−8
3.0	1000	3	0.76	0.001	−2.0E−7	3.5E−9	5.7E−5	1.3E−7
3.45	600	29	0.75	0.015	−1.6E−7	4.1E−9	5.4E−5	2.4E−6
3.8	404	3	0.76	0.003	−6.8E−8	2.8E−9	5.2E−5	3.0E−7
4.4	220	1	0.79	0.003	8.0E−8	4.5E−9	4.5E−5	2.1E−7
5.0	112	5	0.76	0.016	1.1E−7	7.7E−9	3.9E−5	1.4E−6
5.6	63.0	0.9	0.75	0.005	1.8E−7	1.6E−9	3.1E−5	3.6E−7
6.4	36.7	0.5	0.80	0.006	3.0E−7	2.4E−9	1.8E−5	1.8E−7
7.0	23.7	0.7	0.76	0.011	3.0E−7	2.7E−9	1.1E−5	2.2E−7

Table S3b. Relaxation times of $Dy_2LuN@C_{80}$ measured in a field of 0.2 T

T, K	*τ*, s	±	β	±	M_{eq}	±	M_0	±
1.8	2319	8	0.61	0.002	4.9E−5	3.5E−8	7.2E−5	5.7E−8
1.9	2049	6	0.61	0.002	4.7E−5	2.9E−8	7.1E−5	5.3E−8
2.0	1786	4	0.62	0.001	4.5E−5	2.2E−8	7.1E−5	4.5E−8
2.2	1428	2	0.63	0.001	4.2E−5	1.5E−8	7.0E−5	3.4E−8
2.5	1076	5	0.62	0.002	3.8E−5	6.4E−9	6.9E−5	8.0E−8
3.0	677	2	0.66	0.001	3.2E−5	6.9E−9	6.5E−5	5.9E−8
3.8	322	1	0.70	0.002	2.5E−5	6.8E−9	5.9E−5	7.5E−8
4.4	185	1	0.71	0.003	2.1E−5	6.4E−9	5.2E−5	1.4E−7
5.0	110	1	0.77	0.002	1.9E−5	5.6E−9	4.5E−5	6.9E−8
5.6	64.8	0.5	0.74	0.004	1.6E−5	3.7E−9	3.7E−5	1.0E−7
6.4	37.6	0.5	0.74	0.007	1.4E−5	3.2E−9	2.7E−5	9.7E−8

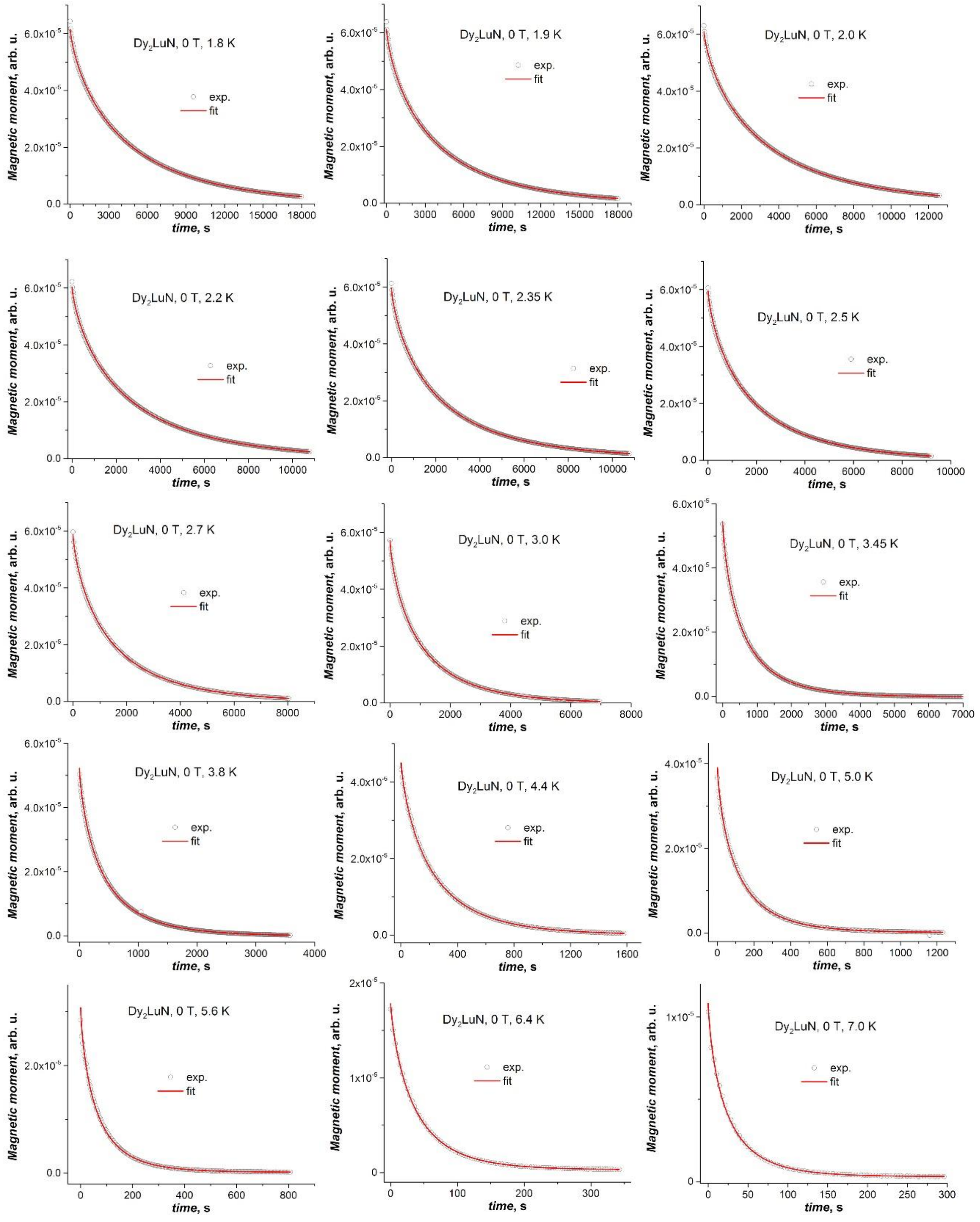

Figure S3. Selected magnetization decay curves of $Dy_2LuN@C_{80}$ measured in zero field at different temperatures. The complete set of determined values is listed in Table S3a.

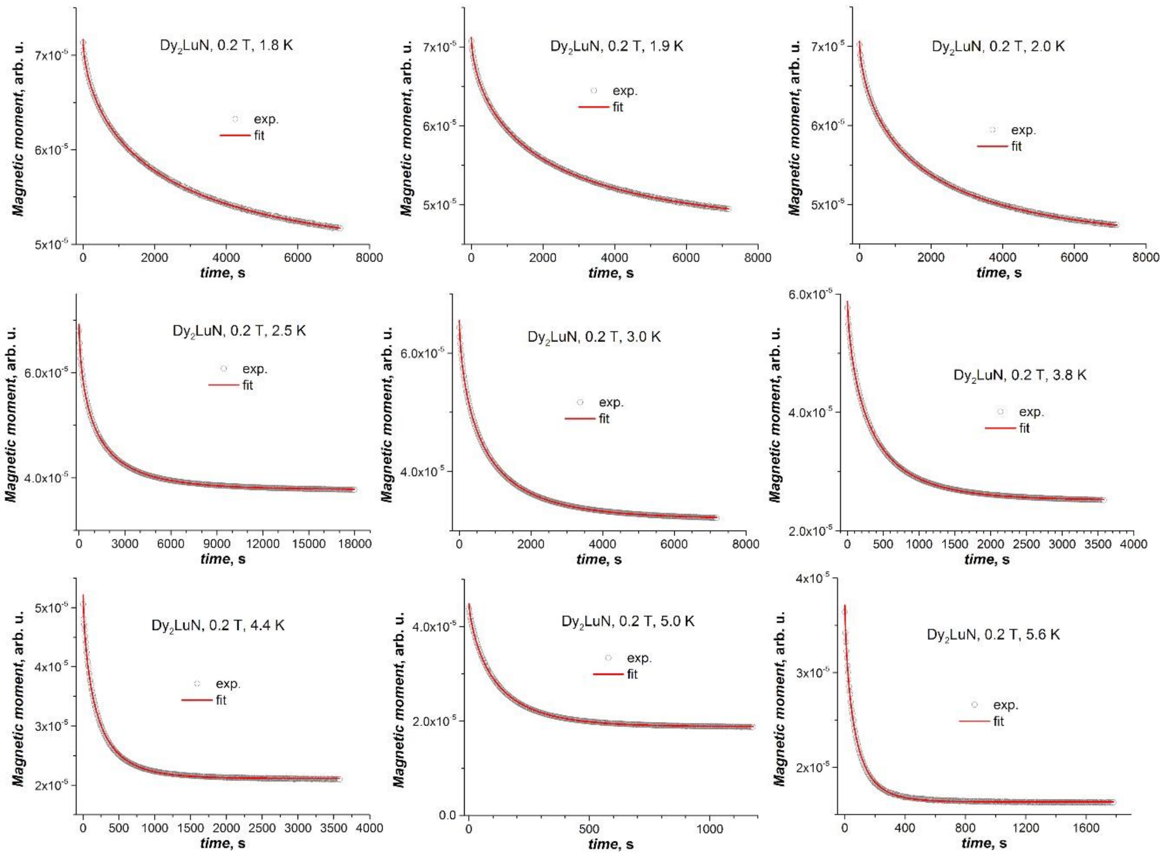

Figure S4. Selected magnetization decay curves of $Dy_2LuN@C_{80}$ measured at different temperatures in a field of 0.2 T. The complete set of determined values is listed in Table S3b.

Table S4. Relaxation times of $Dy_2LuN@C_{80}$ measured at 2.5 K in different magnetic fields

H, **T**	***τ*, s**	**±**	**β**	**±**	***M_{eq}***	**±**	***M₀***	**±**
0.00	1748	2	0.76	0.001	−2.9E−7	5.2E−9	5.9E−5	6.7E−8
0.025	1667	2	0.73	0.001	4.6E−6	1.4E−8	6.0E−5	6.6E−8
0.05	1625	2	0.72	0.001	9.7E−6	1.8E−8	6.2E−5	7.3E−8
0.10	1564	2	0.70	0.001	2.0E−5	1.4E−8	6.5E−5	4.8E−8
0.15	1367	1	0.68	0.001	2.9E−5	1.0E−8	6.7E−5	3.5E−8
0.20	1076	5	0.62	0.002	3.8E−5	6.4E−9	6.9E−5	8.0E−8
0.25	786	3	0.61	0.002	4.5E−5	1.1E−8	6.9E−5	5.6E−8
0.30	593	4	0.58	0.003	5.0E−5	1.1E−8	7.0E−5	7.3E−8
0.40	400	5	0.52	0.004	5.9E−5	1.0E−8	7.2E−5	7.7E−8
0.50	359	5	0.49	0.004	6.5E−5	8.8E−9	7.3E−5	6.0E−8

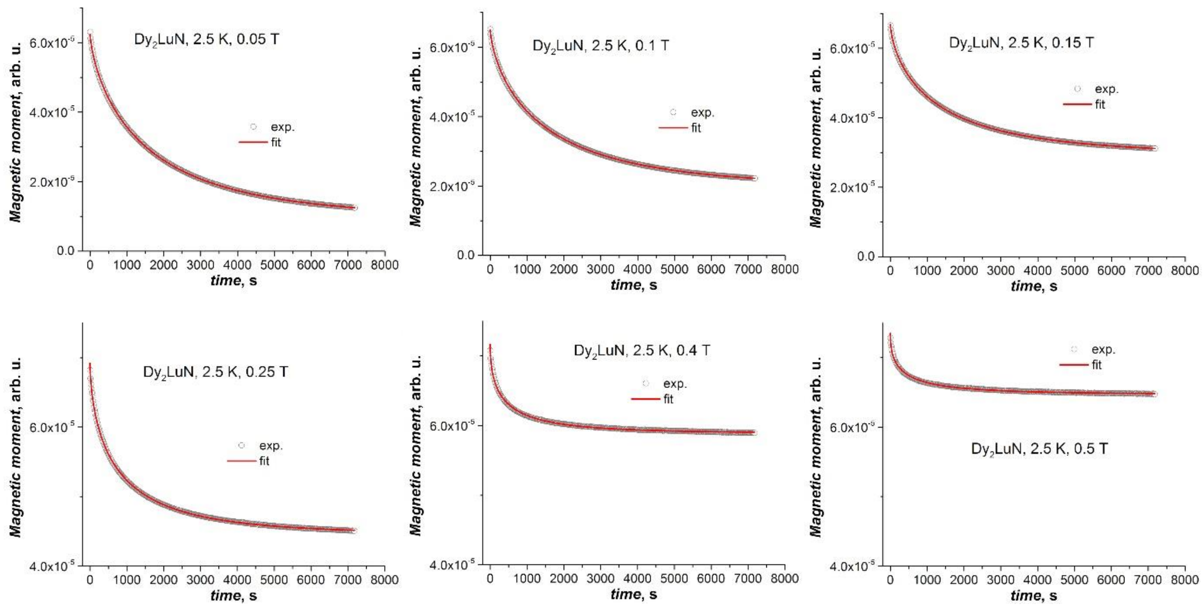

Figure S5. Selected magnetization decay curves of $Dy_2LuN@C_{80}$ measured at 2.5 K in different magnetic fields. Dots – experimental data, red lines – fits with stretched exponentials. The complete set of determined values is listed in Table S4.

Table S5a. Relaxation times of $Dy_2ScN@C_{80}$ measured in zero field

T, K	*τ*, s	±	β	±	M_{eq}	±	M_0	±
1.8	4426	2.2	0.87	0.001	−1.7E−6	7.8E−8	5.3E−4	3.2E−7
1.9	3492	1.7	0.87	0.001	−2.0E−6	8.4E−8	5.4E−4	3.2E−7
2.0	2753	1.3	0.86	0.001	−1.9E−6	6.5E−8	5.4E−4	2.9E−7
2.1	2257	1.1	0.86	0.001	−1.5E−6	1.0E−7	5.4E−4	3.2E−7
2.2	1886	0.9	0.86	0.001	−1.6E−6	7.7E−8	5.3E−4	3.1E−7
2.35	1470	0.7	0.86	0.001	−1.5E−6	6.4E−8	5.3E−4	2.7E−7
2.5	1180	0.5	0.86	0.000	−1.5E−6	4.3E−8	5.1E−4	2.4E−7
2.7	898	0.4	0.85	0.000	−1.7E−6	2.8E−8	5.1E−4	2.2E−7
3.0	622	0.4	0.85	0.000	−2.0E−6	1.6E−8	5.0E−4	2.6E−7
3.45	367	0.8	0.83	0.001	−2.5E−6	6.6E−9	4.8E−4	9.7E−7
3.8	270	0.2	0.86	0.001	−1.2E−6	2.0E−8	4.3E−4	2.1E−7
4.4	151	0.3	0.87	0.001	−1.0E−6	1.8E−8	3.7E−4	5.3E−7
5.0	86.1	0.4	0.86	0.002	−1.2E−6	9.0E−9	3.1E−4	1.4E−6
5.6	49.8	0.8	0.84	0.005	−1.4E−6	7.5E−9	2.4E−4	3.7E−6
6.4	27.7	0.3	0.83	0.004	−1.0E−6	2.9E−9	1.4E−4	1.8E−6
7.0	19.6	0.3	0.84	0.006	−9.7E−7	3.3E−9	8.4E−5	1.3E−6

Table S5b. Relaxation times of $Dy_2ScN@C_{80}$ measured in a field of 0.2 T

T, K	*τ*, s	±	β	±	M_{eq}	±	M_0	±
1.8	3990	9.5	0.82	0.003	3.8E-04	1.8E-07	5.5E−4	3.9E−7
1.9	3191	7.5	0.81	0.003	3.7E-04	2.0E-07	5.5E−4	4.1E−7
2.0	2638	8.5	0.79	0.003	3.5E-04	3.2E-07	5.5E−4	5.2E−7
2.2	1847	4.4	0.79	0.002	3.3E-04	2.6E-07	5.4E−4	4.5E−7
2.5	1166	1.7	0.80	0.002	3.0E-04	1.5E-07	5.3E−4	3.3E−7
3.0	628	0.8	0.80	0.001	2.5E-04	1.7E-07	2.6E−4	5.1E−4
3.45	373	0.2	0.83	0.001	2.3E-04	3.7E-08	4.9E−4	1.4E−7
3.8	256	0.2	0.84	0.001	2.0E-04	4.1E-08	4.6E−4	1.5E−7
4.4	142	0.1	0.87	0.001	1.8E-04	1.7E-08	4.1E−4	1.7E−7
5.0	82.6	0.2	0.88	0.002	1.5E-04	2.6E-08	3.4E−4	3.8E−7
5.6	51.8	0.2	0.90	0.003	1.3E-04	1.9E-08	2.8E−4	3.3E−7
6.4	29.0	0.3	0.91	0.008	1.2E-04	3.5E-08	2.0E−4	6.2E−7
7.0	20.2	0.2	0.90	0.009	1.1E-04	3.0E-08	1.6E−4	3.0E−7

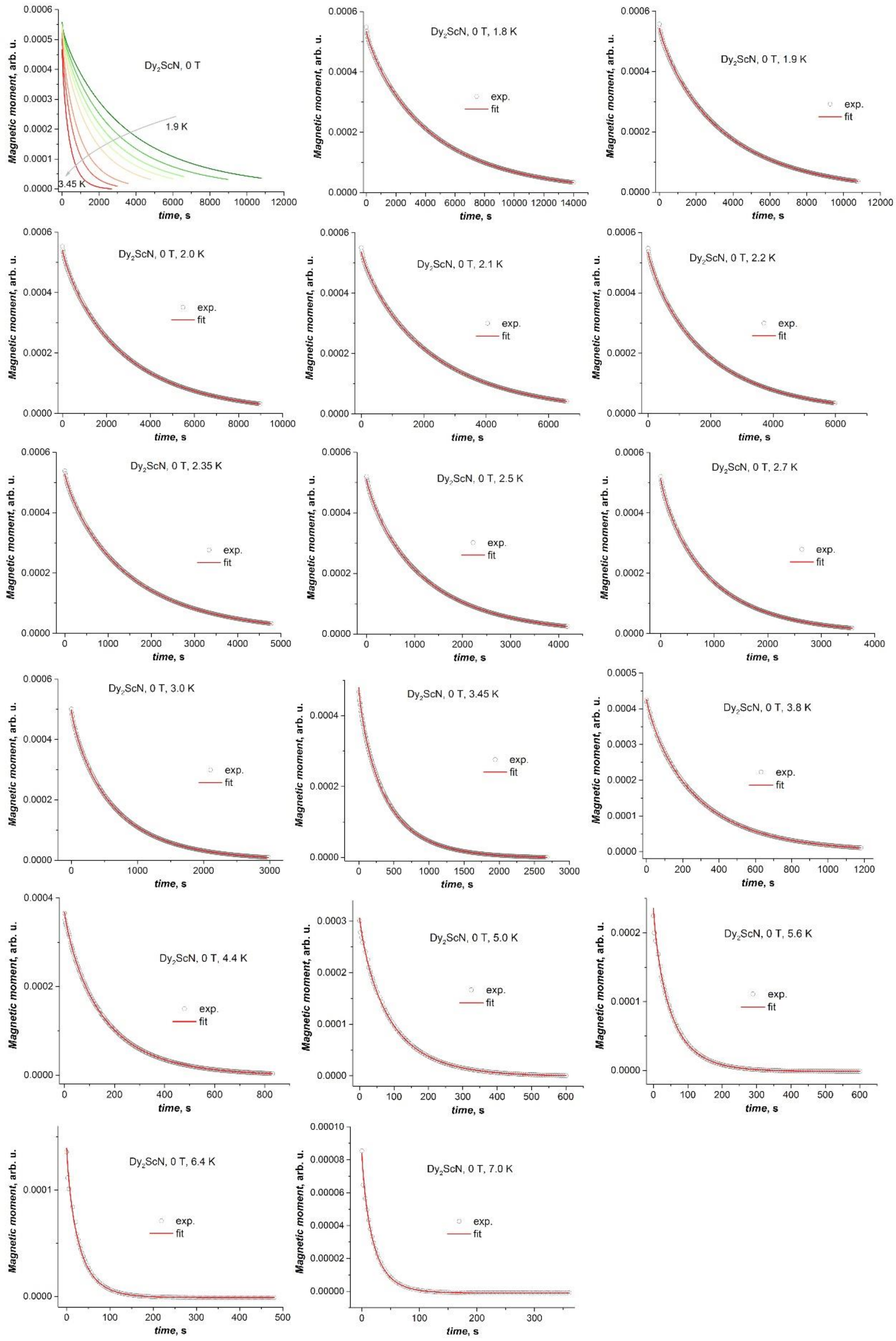

Figure S6. Selected magnetization decay curves of $Dy_2ScN@C_{80}$ measured at different temperatures in zero magnetic field. The complete set of determined values is listed in Table S5a.

Table S6. Relaxation times of $Dy_2ScN@C_{80}$ measured at 2.5 K in different magnetic fields

***H*, T**	***τ*, s**	**±**	**β**	**±**	***M***	**±**	***M*₀**	**±**
0.00	1180	0.5	0.86	0.000	−1.5E−6	4.3E−8	5.1E−4	2.4E−7
0.025	1174	0.7	0.86	0.001	4.2E−5	7.2E−8	5.1E−4	2.9E−7
0.05	1170	0.8	0.85	0.001	8.4E−5	9.1E−8	5.2E−4	3.1E−7
0.10	1164	0.9	0.84	0.001	1.7E−4	1.0E−7	5.2E−4	2.9E−7
0.20	1166	1.7	0.80	0.002	3.0E−4	1.5E−7	5.3E−4	3.3E−7
0.25	1173	2.1	0.77	0.002	3.5E−4	1.5E−7	5.4E−4	3.2E−7
0.30	1181	2.4	0.74	0.002	3.9E−4	1.4E−7	5.4E−4	2.7E−7
0.40	1179	2.8	0.68	0.002	4.6E−4	9.8E−8	5.5E−4	1.8E−7
0.50	1050	2.7	0.62	0.002	5.0E−4	6.3E−8	5.6E−4	1.2E−7

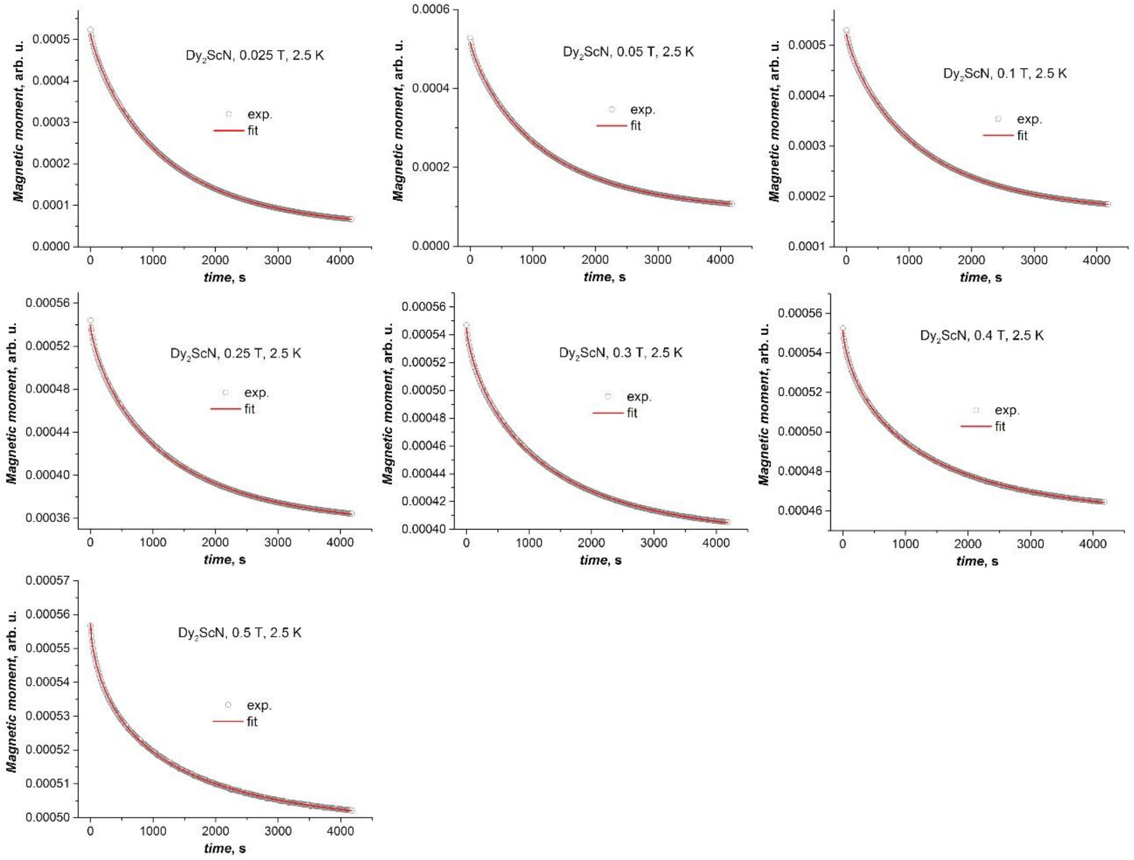

Figure S7. Magnetization decay curves of $Dy_2ScN@C_{80}$ measured at 2.5 K in different magnetic fields. Dots – experimental data, red lines – fits with stretched exponentials. The complete set of determined values is listed in Table S6.

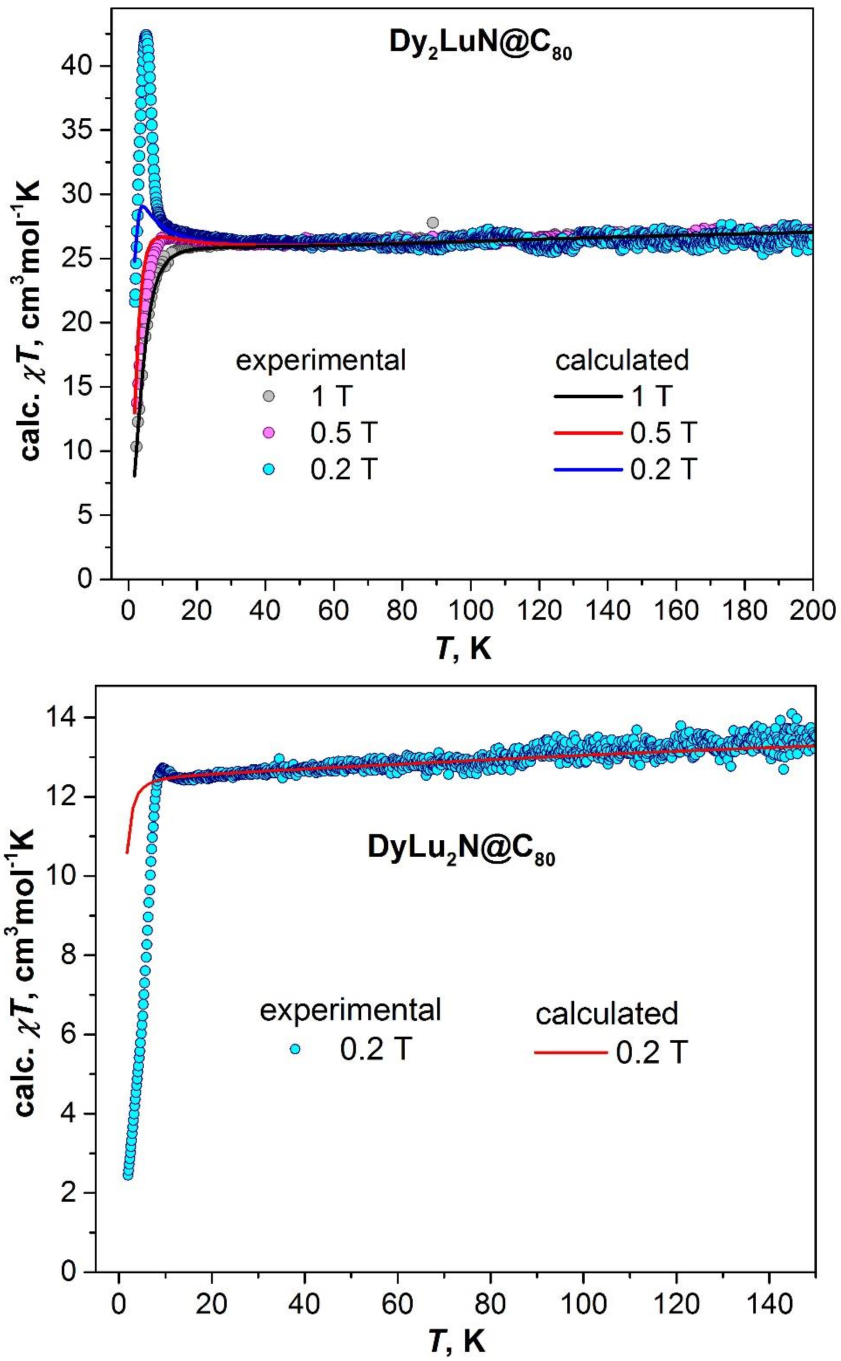

Figure S8. Experimental (dots) and calculated (lines) χT curves for $Dy_2LuN@C_{80}$ and $DyLu_2N@C_{80}$. Note that due to the long relaxation times below TB, experimental χT values at these temperatures do not correspond to thermodynamic equilibrium and hence can deviate strongly from simulated curves.

DFT-optimized atomic coordinates

$DyLu_2N@C_{80}$

C	0.948410000	2.244587000	-3.312715000
C	2.085982000	2.523598000	-2.479062000
C	2.951999000	1.363754000	-2.481619000
C	1.099430000	0.912163000	-3.833318000
C	2.329599000	0.371461000	-3.324430000
C	-0.034742000	0.074466000	-4.048583000
C	-1.327148000	0.639542000	-3.831739000
C	-0.342080000	2.779008000	-3.015645000
C	-1.474451000	1.975513000	-3.322546000
C	-0.468379000	3.693780000	-1.900814000
C	1.960053000	3.341011000	-1.328693000
C	0.671402000	3.937760000	-1.062110000
C	2.792616000	3.031202000	-0.215424000
C	3.778597000	1.013177000	-1.359188000
C	3.679166000	1.885540000	-0.219473000
C	4.074548000	-0.397324000	-1.112547000
C	2.458586000	-1.010086000	-3.014256000
C	3.312117000	-1.358060000	-1.898383000
C	0.124097000	-1.327885000	-3.832370000
C	1.353069000	-1.863052000	-3.311499000
C	-0.957951000	-2.122140000	-3.322471000
C	-2.404930000	-0.157194000	-3.311532000
C	-2.218354000	-1.541790000	-3.014112000
C	-2.647056000	2.021288000	-2.479768000
C	-3.215797000	0.688882000	-2.478501000
C	-1.682447000	3.870850000	-1.112987000
C	-2.767003000	2.921432000	-1.362211000
C	0.198698000	4.219065000	0.270154000
C	-1.268140000	4.224392000	0.271346000
C	2.318751000	3.236116000	1.121727000
C	1.015120000	3.778946000	1.384167000
C	3.755514000	1.371975000	1.139832000
C	2.886044000	2.207129000	1.951248000
C	4.189262000	-0.935871000	0.271206000
C	3.969484000	-0.034772000	1.418495000
C	2.957983000	-2.468861000	-1.059463000
C	3.448936000	-2.205879000	0.270753000
C	1.027890000	-2.988025000	-2.477621000
C	-0.410681000	-3.159614000	-2.478249000
C	1.798155000	-3.289336000	-1.327497000
C	-1.129884000	-3.718240000	-1.364023000
C	-2.942747000	-2.109400000	-1.897845000
C	-2.497887000	-3.257042000	-1.115696000
C	-3.854421000	0.171973000	-1.325404000

C	-3.716347000	-1.240474000	-1.056644000
C	-3.472898000	2.399531000	-0.217925000
C	-4.005870000	1.049912000	-0.213281000
C	-1.941511000	3.596964000	1.421185000
C	-3.063292000	2.719563000	1.144621000
C	0.332215000	3.306614000	2.535324000
C	-1.113013000	3.165918000	2.523034000
C	2.155824000	1.686552000	3.059224000
C	0.892257000	2.279485000	3.370446000
C	3.180122000	-0.534680000	2.518992000
C	2.341251000	0.305768000	3.350552000
C	2.651604000	-2.686679000	1.382676000
C	2.580111000	-1.856671000	2.532101000
C	1.114000000	-3.858552000	-0.215035000
C	-0.322429000	-4.066228000	-0.219650000
C	1.528926000	-3.544834000	1.120548000
C	-0.802546000	-3.867492000	1.142703000
C	0.354393000	-3.521358000	1.950716000
C	-3.007043000	-3.069646000	0.272140000
C	-3.724553000	-1.791454000	0.272677000
C	-2.122885000	-3.333542000	1.420128000
C	-3.941448000	0.534442000	1.122476000
C	-3.339303000	1.542239000	1.953021000
C	-3.757027000	-0.865223000	1.385792000
C	-1.419993000	2.018581000	3.355169000
C	-0.190710000	1.480035000	3.868117000
C	-2.522009000	1.167616000	3.061357000
C	1.262262000	-0.489433000	3.865429000
C	1.412449000	-1.827528000	3.368253000
C	0.268294000	-2.626808000	3.058847000
C	-0.031545000	0.076070000	4.086436000
C	-2.163482000	-2.400399000	2.519979000
C	-3.009967000	-1.221071000	2.536815000
C	-2.401514000	-0.223769000	3.371809000
C	-1.018014000	-2.095642000	3.352533000
C	-1.166963000	-0.762846000	3.867907000
N	0.000000000	0.000000000	0.000000000
Dy	-0.887663000	1.891872000	0.017488000
Lu	2.027411000	-0.171023000	0.034333000
Lu	-1.199106000	-1.645730000	-0.007744000

$DySc_2N@C_{80}$

C	0.895621000	2.361703000	-3.307101000
C	2.032213000	2.642802000	-2.471329000
C	2.891296000	1.482786000	-2.476161000
C	1.046114000	1.028791000	-3.829933000
C	2.274787000	0.487994000	-3.320317000
C	-0.087983000	0.190002000	-4.045721000
C	-1.380954000	0.756625000	-3.829016000
C	-0.393748000	2.890907000	-3.006325000
C	-1.527430000	2.091191000	-3.316624000
C	-0.515178000	3.792627000	-1.882564000
C	1.909875000	3.458530000	-1.316249000
C	0.621978000	4.044195000	-1.045803000
C	2.732953000	3.155140000	-0.196023000
C	3.684579000	1.128475000	-1.337621000
C	3.589059000	1.995403000	-0.200709000
C	3.968744000	-0.274969000	-1.086275000
C	2.399029000	-0.894129000	-3.007635000
C	3.233976000	-1.241850000	-1.880776000
C	0.072170000	-1.214079000	-3.830267000
C	1.301247000	-1.750874000	-3.307382000
C	-1.007884000	-2.006121000	-3.318799000
C	-2.458864000	-0.039083000	-3.307284000
C	-2.264307000	-1.419060000	-3.006729000
C	-2.697999000	2.132160000	-2.470359000
C	-3.270232000	0.805539000	-2.470389000
C	-1.721868000	3.951635000	-1.087983000
C	-2.806441000	3.014962000	-1.340767000
C	0.150761000	4.316006000	0.288965000
C	-1.309664000	4.303261000	0.289588000
C	2.266536000	3.362282000	1.146764000
C	0.964053000	3.893889000	1.409199000
C	3.656301000	1.481498000	1.153020000
C	2.822772000	2.328455000	1.976988000
C	4.078023000	-0.808650000	0.288526000
C	3.855819000	0.082533000	1.428256000
C	2.891049000	-2.356099000	-1.044187000
C	3.365187000	-2.085232000	0.289784000
C	0.979306000	-2.877338000	-2.471231000
C	-0.456030000	-3.042007000	-2.472353000
C	1.744130000	-3.184066000	-1.315913000
C	-1.163258000	-3.580643000	-1.345361000
C	-2.973565000	-1.969456000	-1.877531000
C	-2.514169000	-3.100290000	-1.089712000
C	-3.907716000	0.294860000	-1.312424000
C	-3.753793000	-1.107176000	-1.040005000
C	-3.512102000	2.500944000	-0.197000000

C	-4.058175000	1.162552000	-0.192170000
C	-1.981210000	3.688154000	1.438313000
C	-3.102675000	2.820462000	1.164117000
C	0.281588000	3.418532000	2.561468000
C	-1.160356000	3.271552000	2.547292000
C	2.101267000	1.803681000	3.088539000
C	0.839376000	2.396066000	3.402929000
C	3.107922000	-0.420065000	2.547109000
C	2.283458000	0.422067000	3.382975000
C	2.593464000	-2.575466000	1.412371000
C	2.520505000	-1.744660000	2.562763000
C	1.071435000	-3.746499000	-0.193737000
C	-0.364006000	-3.929485000	-0.199099000
C	1.483744000	-3.440474000	1.147885000
C	-0.839811000	-3.723397000	1.160846000
C	0.310551000	-3.404604000	1.978792000
C	-3.008709000	-2.901913000	0.290528000
C	-3.738066000	-1.643350000	0.292131000
C	-2.144568000	-3.172378000	1.435948000
C	-3.997138000	0.652117000	1.148422000
C	-3.391567000	1.655370000	1.979666000
C	-3.801965000	-0.739727000	1.415478000
C	-1.471484000	2.131764000	3.385719000
C	-0.243330000	1.597033000	3.904146000
C	-2.575643000	1.282898000	3.091832000
C	1.207555000	-0.374621000	3.902362000
C	1.358317000	-1.714602000	3.404196000
C	0.216794000	-2.512775000	3.090720000
C	-0.084878000	0.192243000	4.125280000
C	-2.208577000	-2.270165000	2.551623000
C	-3.063039000	-1.099329000	2.570412000
C	-2.456610000	-0.106154000	3.408880000
C	-1.068364000	-1.977042000	3.388063000
C	-1.220202000	-0.645751000	3.906894000
N	0.000000000	0.000000000	0.000000000
Dy	-0.905834000	1.956045000	0.047942000
Sc	1.970546000	-0.122998000	0.018782000
Sc	-1.202171000	-1.573150000	-0.020437000

$Dy_2LuN@C_{80}$

C	1.029114000	2.235967000	-3.276844000
C	2.168011000	2.515051000	-2.445361000
C	3.038512000	1.356274000	-2.446756000
C	1.180282000	0.903765000	-3.795419000
C	2.411604000	0.363214000	-3.287546000
C	0.045391000	0.067298000	-4.011229000
C	-1.247487000	0.632235000	-3.794958000
C	-0.262580000	2.770741000	-2.980639000
C	-1.395333000	1.967959000	-3.286781000
C	-0.392036000	3.687032000	-1.868024000
C	2.038786000	3.329733000	-1.294712000
C	0.748246000	3.924605000	-1.028322000
C	2.876058000	3.018330000	-0.184191000
C	3.880729000	1.009483000	-1.331258000
C	3.781437000	1.883149000	-0.188150000
C	4.166843000	-0.405289000	-1.083109000
C	2.542472000	-1.019118000	-2.980816000
C	3.402485000	-1.368282000	-1.869241000
C	0.203966000	-1.334221000	-3.796171000
C	1.433551000	-1.869663000	-3.276784000
C	-0.878819000	-2.129326000	-3.288036000
C	-2.325357000	-0.164545000	-3.275852000
C	-2.139792000	-1.549939000	-2.979639000
C	-2.570131000	2.016475000	-2.445261000
C	-3.137468000	0.681619000	-2.444703000
C	-1.609829000	3.873704000	-1.084678000
C	-2.700404000	2.926462000	-1.334914000
C	0.274569000	4.204673000	0.302000000
C	-1.192793000	4.221452000	0.303122000
C	2.397401000	3.220832000	1.150754000
C	1.093158000	3.765836000	1.413831000
C	3.857885000	1.369212000	1.174789000
C	2.971862000	2.196473000	1.981008000
C	4.273582000	-0.942729000	0.302591000
C	4.064136000	-0.041858000	1.452973000
C	3.045213000	-2.478590000	-1.030417000
C	3.530780000	-2.211741000	0.300934000
C	1.107273000	-2.994778000	-2.444162000
C	-0.331690000	-3.167298000	-2.445648000
C	1.880311000	-3.294965000	-1.295661000
C	-1.052610000	-3.724753000	-1.331727000
C	-2.867908000	-2.119574000	-1.866044000
C	-2.424934000	-3.270032000	-1.085069000
C	-3.778249000	0.163895000	-1.293671000
C	-3.644239000	-1.251338000	-1.025422000
C	-3.408497000	2.403332000	-0.188155000

C	-3.929797000	1.045865000	-0.183630000
C	-1.869170000	3.597346000	1.454836000
C	-2.997330000	2.722452000	1.177599000
C	0.410616000	3.298247000	2.565060000
C	-1.036089000	3.159918000	2.552177000
C	2.237324000	1.677029000	3.088076000
C	0.971914000	2.269947000	3.397421000
C	3.268811000	-0.543030000	2.551495000
C	2.424586000	0.296927000	3.380723000
C	2.733873000	-2.694366000	1.412793000
C	2.664698000	-1.864404000	2.562193000
C	1.193045000	-3.863037000	-0.184305000
C	-0.243950000	-4.072171000	-0.188720000
C	1.608116000	-3.550362000	1.150708000
C	-0.726219000	-3.878329000	1.173455000
C	0.432564000	-3.529180000	1.979902000
C	-2.941742000	-3.090026000	0.303648000
C	-3.658575000	-1.806593000	0.303291000
C	-2.051767000	-3.352461000	1.452503000
C	-3.861854000	0.527732000	1.150210000
C	-3.261778000	1.537445000	1.980802000
C	-3.681087000	-0.874563000	1.413418000
C	-1.341267000	2.010824000	3.382687000
C	-0.111505000	1.471078000	3.894314000
C	-2.442990000	1.160569000	3.088120000
C	1.342719000	-0.497455000	3.892056000
C	1.493310000	-1.834813000	3.395010000
C	0.348021000	-2.633567000	3.086135000
C	0.047980000	0.067597000	4.111168000
C	-2.087285000	-2.412032000	2.548530000
C	-2.931753000	-1.229726000	2.563217000
C	-2.322059000	-0.231299000	3.396249000
C	-0.939584000	-2.103629000	3.378238000
C	-1.087827000	-0.770689000	3.891921000
N	0.000000000	0.000000000	0.000000000
Dy	-0.870664000	1.882309000	0.038440000
Dy	2.067988000	-0.125072000	0.059732000
Lu	-1.158992000	-1.654370000	0.040187000

$Dy_2ScN@C_{80}$

C	1.104602000	2.343668000	-3.301642000
C	2.242673000	2.622166000	-2.468054000
C	3.108850000	1.463478000	-2.469953000
C	1.255413000	1.011501000	-3.821075000
C	2.486402000	0.470332000	-3.311486000
C	0.120320000	0.175899000	-4.037352000
C	-1.173527000	0.741768000	-3.821411000
C	-0.186751000	2.875711000	-3.003641000
C	-1.320800000	2.076047000	-3.310866000
C	-0.315113000	3.781960000	-1.885323000
C	2.113923000	3.433488000	-1.314449000
C	0.823680000	4.018389000	-1.046348000
C	2.946309000	3.125680000	-0.200273000
C	3.929384000	1.112136000	-1.343282000
C	3.834611000	1.983370000	-0.204041000
C	4.216585000	-0.296544000	-1.093539000
C	2.616191000	-0.911638000	-3.002037000
C	3.468837000	-1.262131000	-1.885693000
C	0.280515000	-1.226233000	-3.821793000
C	1.510202000	-1.762464000	-3.300140000
C	-0.801204000	-2.020071000	-3.314097000
C	-2.251867000	-0.054402000	-3.302251000
C	-2.060618000	-1.437053000	-3.004411000
C	-2.495427000	2.123664000	-2.466717000
C	-3.064344000	0.790996000	-2.468663000
C	-1.529935000	3.962330000	-1.100055000
C	-2.628310000	3.031363000	-1.354558000
C	0.350962000	4.286189000	0.284753000
C	-1.110305000	4.297932000	0.285102000
C	2.472992000	3.329926000	1.137528000
C	1.169164000	3.867550000	1.400757000
C	3.913661000	1.469819000	1.155658000
C	3.043373000	2.304637000	1.968027000
C	4.336620000	-0.834488000	0.285546000
C	4.126659000	0.064576000	1.433969000
C	3.122840000	-2.377123000	-1.049385000
C	3.610607000	-2.112524000	0.283986000
C	1.186421000	-2.888422000	-2.467128000
C	-0.250632000	-3.057199000	-2.471100000
C	1.959381000	-3.192988000	-1.316664000
C	-0.964387000	-3.598428000	-1.349165000
C	-2.781714000	-1.996904000	-1.884139000
C	-2.329992000	-3.138299000	-1.100324000
C	-3.703907000	0.278028000	-1.314691000
C	-3.560318000	-1.131344000	-1.044484000
C	-3.338566000	2.513850000	-0.204884000

C	-3.854813000	1.155507000	-0.199911000
C	-1.787771000	3.690152000	1.437387000
C	-2.925073000	2.830225000	1.162941000
C	0.486965000	3.404227000	2.553737000
C	-0.957914000	3.261327000	2.537995000
C	2.311543000	1.785635000	3.076674000
C	1.047329000	2.378985000	3.389380000
C	3.340373000	-0.435819000	2.537865000
C	2.498878000	0.404802000	3.368735000
C	2.815143000	-2.591869000	1.398767000
C	2.738702000	-1.756775000	2.546903000
C	1.278134000	-3.756406000	-0.201157000
C	-0.159037000	-3.941117000	-0.206282000
C	1.693080000	-3.449265000	1.137430000
C	-0.636552000	-3.738829000	1.151380000
C	0.517481000	-3.416764000	1.966585000
C	-2.835913000	-2.948839000	0.284973000
C	-3.559545000	-1.677162000	0.285697000
C	-1.951708000	-3.204848000	1.428519000
C	-3.786398000	0.638847000	1.135908000
C	-3.186817000	1.647119000	1.967078000
C	-3.600873000	-0.758919000	1.403266000
C	-1.265312000	2.117954000	3.373792000
C	-0.035996000	1.580228000	3.888589000
C	-2.368196000	1.270068000	3.077527000
C	1.418793000	-0.389791000	3.882219000
C	1.569089000	-1.726476000	3.383072000
C	0.425019000	-2.525174000	3.074614000
C	0.123814000	0.176406000	4.104301000
C	-2.006668000	-2.291638000	2.539381000
C	-2.857764000	-1.116056000	2.555787000
C	-2.248047000	-0.120041000	3.389375000
C	-0.862072000	-1.992680000	3.369583000
C	-1.011594000	-0.660151000	3.884582000
N	0.000000000	0.000000000	0.000000000
Dy	-0.848524000	1.929650000	0.015692000
Dy	2.101301000	-0.090586000	0.075464000
Sc	-1.105534000	-1.577807000	-0.023465000

Ab initio calculations of ligand-field splitting

CASSCF calculations, results can be slightly different from earlier reports as they used different molecular geometries

Table S7. Energies and composition in $|m_J\rangle$ basis of the ligand-field states of Dy ions in $DySc_2N@C_{80}$ and $DyLu_2N@C_{80}$.

KD	*E*, cm^{-1}	**$DySc_2N$,** Composition, %[a]	*E*, cm^{-1}	**$DyLu_2N$,** Composition, %[a]
1	0	$99.6\|15/2\rangle$	0	$99.6\|15/2\rangle$
2	356	$96.2\|13/2\rangle + 3.4\|11/2\rangle$	391	$95.9\|13/2\rangle + 3.6\|11/2\rangle$
3	666	$89.1\|11/2\rangle + 6.7\|9/2\rangle + 3.1\|13/2\rangle$	715	$88.3\|11/2\rangle + 7.3\|9/2\rangle + 3.4\|13/2\rangle$
4	906	$82.6\|9/2\rangle + 5.7\|7/2\rangle + 5.0\|5/2\rangle$	960	$81.2\|9/2\rangle + 6.4\|7/2\rangle + 5.5\|11/2\rangle$
5	1050	$65.2\|7/2\rangle + 16.0\|3/2\rangle + 6.2\|-7/2\rangle$	1104	$67.1\|7/2\rangle + 16.2\|3/2\rangle + 4.3\|-1/2\rangle$
6	1140	$53.5\|5/2\rangle + 19.8\|1/2\rangle + 10.6\|-7/2\rangle$	1194	$54.0\|5/2\rangle + 18.8\|1/2\rangle + 11.3\|-7/2\rangle$
7	1222	$32.2\|3/2\rangle + 19.3\|1/2\rangle + 18.7\|-5/2\rangle$	1276	$38.9\|3/2\rangle + 20.4\|1/2\rangle + 18.3\|-5/2\rangle$
8	1284	$43.4\|1/2\rangle + 27.3\|3/2\rangle + 10.4\|5/2\rangle$	1348	$41.3\|1/2\rangle + 30.1\|3/2\rangle + 10.9\|-1/2\rangle$

[a] only 3 largest components are listed

Table S8. Energies and composition in $|m_J\rangle$ basis of the ligand-field states of two Dy ions in $Dy_2LuN@C_{80}$.

KD	E, cm^{-1}	Composition, %[a]	E, cm^{-1}	Composition, %[a]
1	0	$99.7\|15/2\rangle$	0	$99.7\|15/2\rangle$
2	408	$95.0\|13/2\rangle + 4.6\|11/2\rangle$	403	$94.0\|13/2\rangle + 5.4\|11/2\rangle$
3	726	$85.3\|11/2\rangle + 9.6\|9/2\rangle + 4.3\|13/2\rangle$	726	$83.4\|11/2\rangle + 10.8\|9/2\rangle + 5.2\|13/2\rangle$
4	967	$77.3\|9/2\rangle + 8.9\|7/2\rangle + 7.2\|11/2\rangle$	971	$75.8\|9/2\rangle + 9.7\|7/2\rangle + 8.3\|11/2\rangle$
5	1109	$60.4\|7/2\rangle + 14.2\|3/2\rangle + 8.5\|-7/2\rangle$	1112	$51.8\|7/2\rangle + 13.9\|3/2\rangle + 16.4\|-7/2\rangle$
6	1203	$54.1\|5/2\rangle + 17.7\|1/2\rangle + 10.9\|-7/2\rangle$	1202	$57.2\|5/2\rangle + 16.9\|1/2\rangle + 11.0\|-7/2\rangle$
7	1283	$41.5\|3/2\rangle + 18.8\|1/2\rangle + 19.1\|-5/2\rangle$	1286	$34.0\|3/2\rangle + 21.9\|1/2\rangle + 14.9\|-5/2\rangle$
8	1365	$47.4\|1/2\rangle + 24.9\|3/2\rangle + 10.2\|5/2\rangle$	1366	$43.0\|1/2\rangle + 28.8\|3/2\rangle + 11.9\|-1/2\rangle$

[a] only 3 largest components are listed

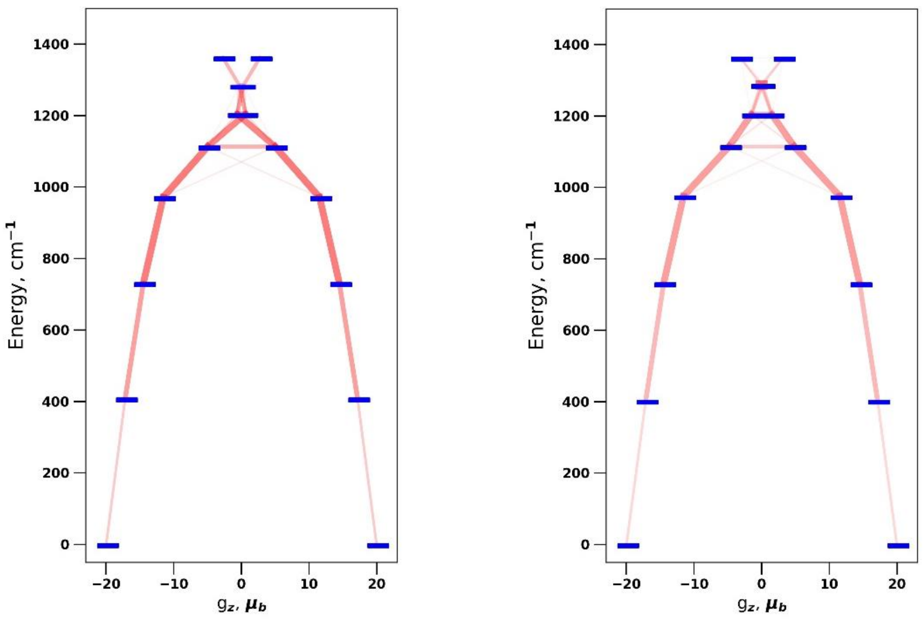

Figure S9. *Ab initio* computed ligand-field states (thick blue dashes) and transition probabilities between them (light blue lines, the thicker the line – the higher the transition probability) for two Dy ions in $Dy_2LuN@C_{80}$ (*ab initio* computations are performed for DFT-optimized atomic coordinates).

Table S9. Energies and composition in $|m_J\rangle$ basis of the ligand-field states of two Dy ions in $Dy_2ScN@C_{80}$.

KD	E, cm^{-1}	Composition, %[a]	E, cm^{-1}	Composition, %[a]
1	0	$99.7\|15/2\rangle$	0	$99.6\|15/2\rangle$
2	399	$93.8\|13/2\rangle + 5.8\|11/2\rangle$	378	$95.4\|13/2\rangle + 4.1\|11/2\rangle$
3	704	$82.0\|11/2\rangle + 12.0\|9/2\rangle + 5.4\|13/2\rangle$	698	$87.2\|11/2\rangle + 8.1\|9/2\rangle + 3.8\|13/2\rangle$
4	938	$71.6\|9/2\rangle + 12.3\|7/2\rangle + 8.7\|11/2\rangle$	945	$80.3\|9/2\rangle + 6.4\|7/2\rangle + 6.1\|11/2\rangle$
5	1081	$57.9\|7/2\rangle + 11.7\|3/2\rangle + 9.1\|-7/2\rangle$	1084	$62.8\|7/2\rangle + 17.5\|3/2\rangle + 6.2\|-7/2\rangle$
6	1176	$54.4\|5/2\rangle + 17.4\|1/2\rangle + 10.0\|-7/2\rangle$	1174	$54.4\|5/2\rangle + 18.4\|1/2\rangle + 11.3\|-7/2\rangle$
7	1253	$32.9\|3/2\rangle + 18.9\|1/2\rangle + 18.4\|-3/2\rangle$	1263	$34.0\|3/2\rangle + 20.3\|1/2\rangle + 18.9\|-5/2\rangle$
8	1336	$45.8\|1/2\rangle + 23.4\|3/2\rangle + 12.9\|-1/2\rangle$	1333	$39.6\|1/2\rangle + 31.1\|3/2\rangle + 12.0\|-1/2\rangle$

[a] only 3 largest components are listed

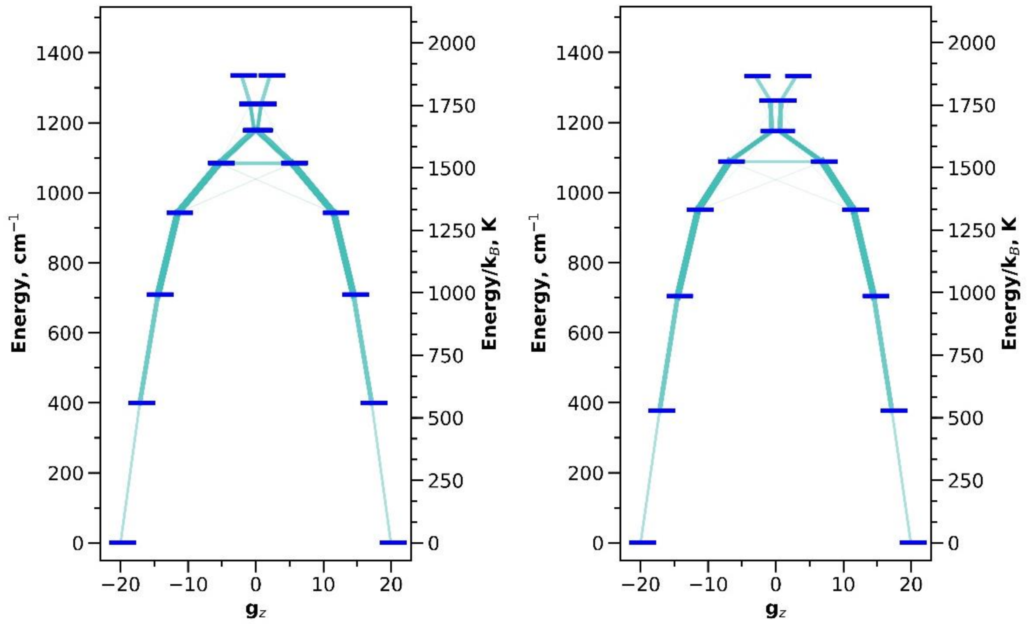

Figure S10. *Ab initio* computed ligand-field states (thick blue dashes) and transition probabilities between them (light blue lines, the thicker the line – the higher the transition probability) for two Dy ions in $Dy_2ScN@C_{80}$ (*ab initio* computations are performed for DFT-optimized atomic coordinates).

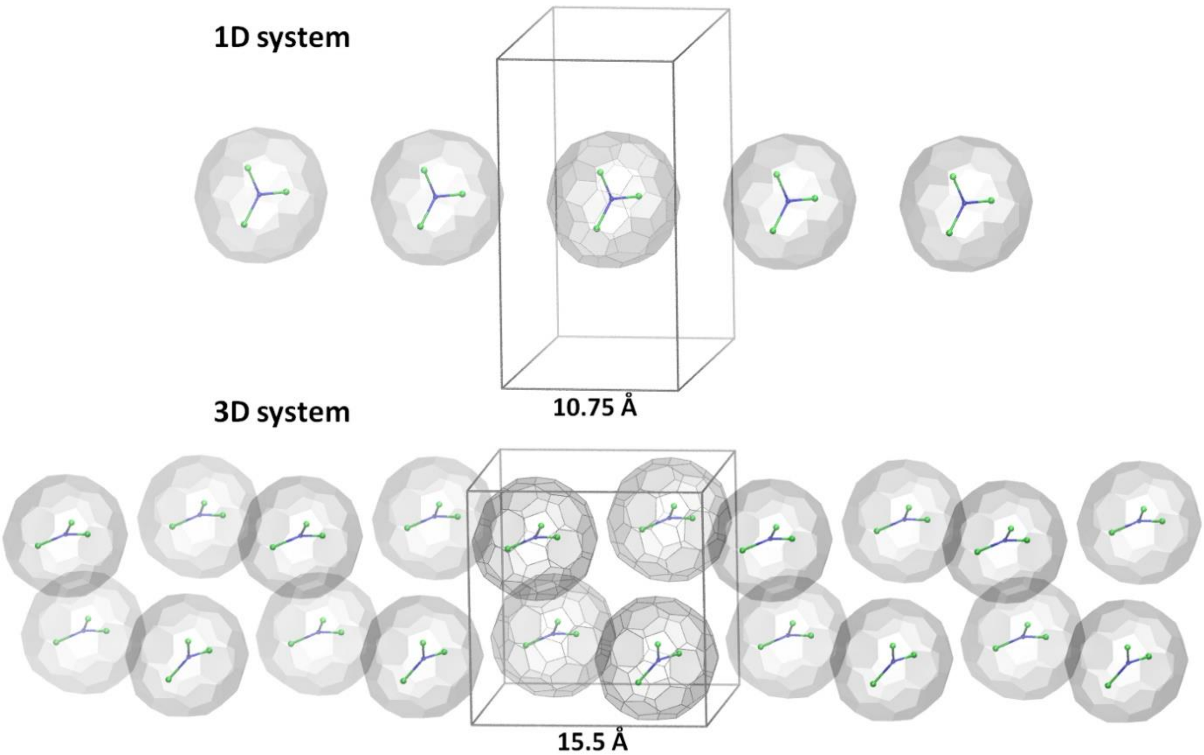

Figure S11. 1D and 3D periodic packing of $Sc_3N@C_{80}$ molecules used in phonon calculations. Box marks periodic cell.
